# Paris saponin VII reverses resistance to PARP inhibitors by regulating ovarian cancer tumor angiogenesis and glycolysis through the RORα/ECM1/VEGFR2 signaling axis

**DOI:** 10.7150/ijbs.91861

**Published:** 2024-04-15

**Authors:** Mengfei Wang, Chenyue Yuan, Zong Wu, Meng Xu, Ziqi Chen, Jialiang Yao, Zujun Que, Jianhui Tian, Elaine Lai-Han Leung, Ziliang Wang

**Affiliations:** 1 Cancer Institute, Shanghai Municipal Hospital of Traditional Chinese Medicine, Shanghai University of Traditional Chinese Medicine, Shanghai, China.; 2 Department of Oncology, Shanghai Municipal Hospital of Traditional Chinese Medicine, Shanghai University of Traditional Chinese Medicine, Shanghai, China.; 3 Cancer Center, Faculty of Health Sciences; MOE Frontiers Science Center for Precision Oncology, University of Macau, Macau (SAR), China.

**Keywords:** Paris saponin VII, PARPi resistance, RORα, ECM1, VEGFR2, glycolysis, angiogenesis, ovarian cancer.

## Abstract

The emergence of Poly (ADP-ribose) polymerase inhibitors (PARPi) has marked the beginning of a precise targeted therapy era for ovarian cancer. However, an increasing number of patients are experiencing primary or acquired resistance to PARPi, severely limiting its clinical application. Deciphering the underlying mechanisms of PARPi resistance and discovering new therapeutic targets is an urgent and critical issue to address. In this study, we observed a close correlation between glycolysis, tumor angiogenesis, and PARPi resistance in ovarian cancer. Furthermore, we discovered that the natural compound Paris saponin VII (PS VII) partially reversed PARPi resistance in ovarian cancer and demonstrated synergistic therapeutic effects when combined with PARPi. Additionally, we found that PS VII potentially hindered glycolysis and angiogenesis in PARPi-resistant ovarian cancer cells by binding and stabilizing the expression of RORα, thus further inhibiting ECM1 and interfering with the VEGFR2/FAK/AKT/GSK3β signaling pathway. Our research provides new targeted treatment for clinical ovarian cancer therapy and brings new hope to patients with PARPi-resistant ovarian cancer, effectively expanding the application of PARPi in clinical treatment.

## Introduction

Ovarian cancer as a common gynecological malignancy ranks third in the incidence of malignant tumors of the female reproductive system according to the International Agency for Research on Cancer (IARC) and has the highest mortality rate 1. Ovarian cancer in the early stages often has no obvious symptoms and lacks effective screening methods. 70% of patients are already in advanced stages (stage III-IV) when diagnosed. Due to the limitations of treatment options, advanced ovarian cancer has a 5-year survival rate as low as 30%, posing a significant threat to women's health and becoming a challenging issue for oncologists and gynecologists in this century 2. The emergence of poly(ADP-ribose) polymerase inhibitors (PARPi) in 2014 has ushered in the era of precision targeted therapy for ovarian cancer, breaking the deadlock in the treatment of advanced ovarian cancer to some extent 3. Due to its remarkable efficacy, PARPi has gradually occupied the stage of maintenance treatment for ovarian cancer 4. The latest version of the NCCN guidelines in 2020 has expanded the recommended range of PARPi to all patients with advanced ovarian cancer, bringing more clinical benefits to patients without genetic mutations 5-7. However, although PARPi has improved patients' survival to some extent and provided a treatment option for advanced ovarian cancer, an increasing number of patients are developing primary or acquired resistance to PARPi, severely limiting its clinical application 8-10. Therefore, in-depth exploration of the mechanisms of PARPi resistance in ovarian cancer and the search for efficient, low-toxicity, and broad-spectrum resistance reversal agents have become significant clinical challenges in the current research field of ovarian cancer tumors.

It is well-known that tumor cells tend to rely on glycolysis rather than oxidative phosphorylation to generate energy, even under aerobic conditions 11. Tumor cells utilize glycolysis to produce a sufficient quantity of metabolic intermediates to sustain the nutritional and energy requirements for rapid growth and proliferation. This unique aerobic glycolysis phenomenon in tumor cells is known as the Warburg effect and is the most important pathway for tumor energy metabolism. During the process of energy acquisition, tumor cells regulate mitochondrial function and control key enzymes that govern metabolism, angiogenesis, and other essential functions 12. The local acidic environment promotes immune evasion, enhances invasiveness, increases tolerance to tumor therapeutic drugs, and impacts various biological characteristics of the tumor. Current research has discovered that glycolysis plays a crucial regulatory role in ovarian cancer resistance 13. Through high-throughput stimulated Raman scattering imaging and single-cell analysis, it has been observed that cisplatin-resistant cells exhibit significant energy metabolism reprogramming. Inhibiting glycolysis can significantly enhance the resistance of ovarian cancer cells to platinum drugs 14. Additionally, there have been studies that found an increased reliance on glycolysis in cisplatin-resistant cells. In our previous research, we also found that platinum resistance could be reversed by regulating the Fibrillin-1/VEGFR2/STAT2 signaling pathway and the Aurora-A/SOX8/FOXK1 signaling pathway to inhibit glycolysis 15,16. Furthermore, the latest research has found that reprogramming cell glycolysis can drive tumor formation and angiogenesis, and also plays a crucial regulatory role in tumor metastasis and drug resistance 17. Previous studies have recognized the significant role of angiogenesis in tumor proliferation and metastasis. It has been discovered that the process of forming new blood vessels requires sufficient oxygen and nutrients, which are dependent on aerobic glycolysis. Correspondingly, the formation of a large number of new tumor blood vessels provides nutrients and oxygen to tumor cells, promotes their migration and invasion, and hinders the cytotoxic effects of chemotherapy drugs, leading to the development of drug resistance in tumor cells 18. Studies have found that inhibiting angiogenesis can reduce cisplatin resistance in cancer 19. The use of anti-angiogenic drugs has profound clinical significance for the treatment of cancer patients. In our own research, by constructing ovarian cancer PARPi-resistant cell lines and conducting RNA-seq analysis comparing them with PARPi drug-sensitive cell lines, we found that glucose metabolism and angiogenesis play a key role in PARPi resistance. Therefore, in-depth exploration of the aerobic glycolysis, angiogenesis mechanisms, and key signaling pathways involved in ovarian cancer PARPi resistance will help address the critical scientific questions related to PARPi drug resistance in ovarian cancer and hold important clinical value for translational research.

Traditional Chinese medicine (TCM) has a history of thousands of years in the treatment of tumors in Asian countries. In recent years, natural medicines such as TCM have gained increasing attention in the field of tumor treatment due to their unique advantages. TCM can be used as an adjunctive therapy in combination with traditional radiation and chemotherapy methods, effectively improving treatment response and survival rates in patients. Paris saponin VII (PS VII) is an active monomer component of the herb Paris polyphylla, and it has been found to exhibit excellent anti-tumor activity in various types of tumors. Current research has found that PS VII can induce apoptosis and inhibit proliferation in colorectal cancer, lung cancer, breast cancer, and other types of cancer 20. Our previous studies have found that PS VII can promote apoptosis in ovarian cancer cells by inhibiting the glycolytic pathway. Considering the crucial regulatory role of glycolysis in drug resistance, we speculate that PS VII may also play a certain role in PARP inhibitor (PARPi) resistance in ovarian cancer. However, the specific extent of its effects, the underlying mechanisms, and related targets have yet to be elucidated.

In this study, we have demonstrated that PS VII effectively reverses glucose metabolism and angiogenesis in ovarian cancer PARPi-resistant cell lines, thereby overcoming PARPi resistance. PS VII also synergistically enhances the therapeutic effects of PARPi, improving anti-tumor efficacy. Further research has revealed that PS VII can directly interact with the retinoic acid receptor-related orphan receptor alpha (RORα), both *in vitro* and *in vivo*, affecting its resistance to PARPi through the extracellular matrix 1 (ECM1)/vascular endothelial growth factor receptor 2 (VEGFR2) signaling pathway. In conclusion, we have identified and confirmed that the natural compound PS VII can serve as a novel reversal agent for PARPi-resistant ovarian cancer. These findings may provide new clinical treatment strategies for the combined use of PS VII and PARPi, uncovering an unexpected role for PS VII as a potential therapeutic target in ovarian cancer.

## Materials and Methods

### Patients and tissue samples

All tissue samples were obtained from the Fudan University Shanghai Cancer Center. All patients provided informed consent for use of their tissues and the study was approved by the Ethics Committee of Fudan University Shanghai Cancer Center (ID 050432-4-1212B).

### Cell culture and drug

The human ovarian epithelial carcinoma cell lines, HEY and SKOV3, were obtained from the American Type Culture Collection (ATCC) or the Cell Bank of the Chinese Academy of Sciences. SKOV3 cells were cultured in RPMI-1640 medium (Hyclone), while HEY cells and human umbilical vein endothelial cells (HUVECs) were cultured in modified Eagle's medium (Hyclone). Cells were cultured at 37 °C in a 5% CO2 environment.

Paris saponin VII (CAS: 68124-04-9; No. HY-N3584) and Olaparib (No. HY-10162) was obtained from MedChemExpress LLC (Shanghai, China). To establish HEY and SKOV3 PARP inhibitor (PARPi) resistant cell lines, the cells were cultured in medium containing olaparib at a starting concentration of 10% of its half-inhibitory concentration value when they reached nearly logarithmic growth phase (70% to 80% cell density). The concentration of olaparib was increased by 10% every 2 weeks until the cells were able to tolerate the maximum dose, and the cells were maintained under drug pressure for 6 months.

### Primer design

Primers were designed by primer premier 5.0 or Basic Local Alignment Search Tool (https://blast.ncbi.nlm.nih.gov/Blast.cgi) and synthesized by Beijing Genomics Institute.

### Quantitative real time PCR (qRT-PCR)

Tissue and cellular RNA was extracted using Total RNA isolation Kit V2 (Vazyme), and real-time reverse transcriptase-PCR was performed.

### Plasmid construction and viral infection

Recombinant plasmids were constructed with full length human cDNA sequences of RORα, ECM1 and VEGFR2. The cDNA sequences of RORα, ECM1 and VEGFR2 were cloned into pCDH-CMV-MCS-EF1-PURO lentiviral vectors, generating overexpression (OE) recombinant plasmids pCDH/RORα OE, pCDH/ECM1 OE and pCDH/VEGFR2 OE. Transduction of lentiviruses carrying recombinant plasmids was performed, the cells were selected with 1 μg/mL puromycin for two weeks.

### RNA-seq data analysis

RNA-seq analysis was performed as previously described 15. Fragments per kilobase of transcript per million mapped reads (FPKM) was used to calculate the gene expression level. Gene Set Enrichment Analysis (GSEA) was used for gene functional annotation.

### Western blot analysis

Antibodies against VEGFA (ab46154), RORα (ab256799), ECM1 (ab126629), CL (ab85799), GATA1 (ab181544), HK2 (ab227198), GLUT1 (ab115730), LDHA (ab52488), VEGFR2 (ab39638), MMP2 (ab92536), MMP3 (ab52915), MMP9 (ab76003), BCL2 (ab182858), GLUT4 (ab188317), ANG2 (ab155106), E-cadherin (ab40772), PTEN (ab267787), FAK (ab76496), AKT1 (ab179463), GSK3β (ab32391), p-FAK (Tyr925; ab230813), p-AKT1 (Ser473; ab81283) and p-GSK3β (Ser9; ab75814) were obtained from Abcam. Western blot assays were performed according to our previous description. All primary antibodies were used at 1:1000 dilutions and secondary antibodies (12-348; Millipore) were used at 1:10000 dilutions.

### Immunohistochemistry (IHC)

Tumor biopsies were fixed with formalin, embedded in paraffin and cut into 5-μm sections. Samples were then deparaffinized and dehydrated, and subsequently rehydrated with demineralized water. The antigen was extracted by an immunohistochemical method utilizing microwave pretreatment. IHC staining was performed as previously described using following antibodies from Abcam: anti-RORα (ab256799), anti-ECM1 (ab126629) and anti-VEGFR2 (ab39638). Goat anti-rabbit horseradish peroxidase-conjugated antibody was used as secondary antibody (ab205718, Abcam). Proteins were visualized *in situ* with a 3, 3ʹ-diaminobenzidine reaction solution.

### Immunoprecipitation (IP)

Cells were lysed in RIPA lysis buffer (Beyotime). Following the manufacturer's directions, protein A/G immunoprecipitation beads (Bimake) were washed using binding buffer, mixed with 25 μg IgG/ECM1/VEGFR2 antibody and incubated for 15 min. Cell lysate was added and the mixture was incubated for 2 h at room temperature. The bead-antigen-antibody complex was then washed twice with buffer, transferred to a new tube and mixed with loading buffer. The beads were heated at 95 °C for 5 min to separate the magnetic beads from the antigen-antibody complex, and the proteins were resolved by SDS-PAGE.

### Silver staining

IP products were separated by SDS-PAGE and stained using the Fast Silver Stain Kit (Beyotime). Briefly, according to the instructions, the glue was fixed, washed, primed, washed again, silver-dyed and washed again. The gel was then colored, the reaction was terminated and the gel was washed one final time. The gel was photographed and the colored band was excised and sent to the company (Biotree) for mass spectrometry.

### Immunofluorescence (IF)

Primary antibodies against RORα (AF7908), ECM1 (ab126629) and VEGFR2 (ab39638) were purchased from Beyotime and Abcam. Goat secondary antibodies anti-mouse IgG H&L (Alexa Fluro 647; ab150115) and anti-rabbit IgG H&L (Alexa Fluro 488; ab150077) were also purchased from Abcam. DNA dye DAPI (4,6-diamidino-2-phenylindole) was obtained from Molecular Probes and cells were photographed with a Leica SP5 confocal fluorescence microscope.

### Luciferase reporter assay

The human ECM1 gene promoter region was cloned into a pGL3 basic vector as pGL3-ECM1-promoter. HEY/RORα OE and SKOV3/RORα OE cells were transfected with 1000 ng of constructed plasmid and 15 ng of Renilla luciferase plasmid. Luciferase activity was measured 48 h later using a Dual Luciferase Assay Kit (Vazyme). Renilla luciferase was used as a control.

### Chromatin immunoprecipitation (ChIP)

ChIP assays were performed as previously described 15. Briefly, cells were crosslinked with 1% formaldehyde, then glycine was used to quench the cross-linking reaction. The cells were lysed in SDS buffer supplemented with protease inhibitor cocktail. Chromatin DNA was splintered into approximately 300 base-pair fragments by ultrasonication, which were then subjected to IP with 10 μg IgG (ab172730, Abcam), or 10 μg RORα (ab256799, Abcam). Washing with low and high-salt-concentration wash buffers, the DNA fragments were de-crosslinked under high-salt conditions. The QIAquick PCR purification kit (Vazyme) was used to purify DNA, followed by qRT-PCR.

### Fluorescence resonance energy transfer and fluorescence lifetime imaging (FRET-FLIM)

FRET-FLIM experiments were performed as previously described 15. Briefly, donor proteins fused to green fluorescent protein (GFP) and acceptor proteins fused to red fluorescent protein (RFP) were expressed from vectors pCMV3-C-GFPSpark and CMV3-C-RFPSpark, respectively. HEY and SKOV3 cells were transiently transfected to co-express both donor and acceptor. Accumulation of the GFP-tagged and RFP-tagged proteins was estimated before measuring lifetime fluorescence. A tunable white light laser set at 489 nm with a pulsed frequency of 40 MHz was used for excitation, and emission was detected using the SMD GFP/RFP Filter Cube (GFP: 500-550 nm). Lifetime fluorescence was normally an amplitude-weighted mean value using data from a single fit (GFP-fused donor protein only or GFP-fused donor protein with a free RFP acceptor, or with noninteracting RFP-fused acceptor protein), or a biexponential fit (GFP-fused donor protein interacting with RFP-fused acceptor protein). Mean fluorescence lifetimes are presented as means ± SEM based on more than 10 cells from at least three independent experiments.

### Glycolysis analysis

The Glucose Uptake Colorimetric Assay Kit (BioVision), Lactate Colorimetric Assay Kit (BioVision), ATP Assay Kit (Sigma-Aldrich) and Amplite Colorimetric NADPH Assay Kit (AAT Bioquest Inc.) were purchased to examine the glycolysis process in ovarian cancer cells according to the manufacturers' introduction.

### Oxygen consumption rate (OCR) and extracellular acidification rate (ECAR)

Cellular mitochondrial function was measured using the Seahorse XF Cell Mito Stress Test Kit (Agilent) and the Bioscience XF96 Extracellular Flux Analyzer, according to the manufacturers' instructions. Glycolytic capacity was determined using the Glycolysis Stress Test Kit (Agilent) per the manufacturer's instructions. A total of 4 × 10^4^ cells were seeded into 96-well plates and incubated overnight. After washing the cells with Seahorse buffer (DMEM with phenol red containing 25 mmol/L glucose, 2 mmol/L sodium pyruvate and 2 mmol/L glutamine), 175 μL of Seahorse buffer plus 25 μL each of 1 mmol/L oligomycin, 1 mmol/L carbonyl cyanide-4 (trifluoromethoxy) phenylhydrazone (FCCP) and 1 mmol/L rotenone was automatically injected to measure the OCR. Then, 25 μL each of 10 mmol/L glucose, 1 mmol/L oligomycin and 100 mmol/L 2-deoxy-glucose were added to measure the ECAR. The OCR and ECAR values were calculated after normalization to the cell number and were plotted as means ± SEM.

### Cell viability assay

A total of 2 × 10^3^ cells were seeded into 96-well plates and supplemented with 0.2 mL maintenance medium to evaluate the cell proliferation rate. Cell Counting Kit-8 (CCK8) (Topscience) (8%) was added and incubated for 1.5 h, at 6 h, 24 h, 48 h and 72 h moments. Cell viability was measured by absorbance at 450 nm by a microplate reader (Synergy H4, Bio-Tek), indicating the number of cells.

### Colony formation assay

A total of 800 cells were seeded into 6-well plates and supplemented with 2 mL maintenance medium to evaluate the cell colony formation rate. After culture for 2 weeks at 37 °C in a 5% CO_2_ humidified environment, the cells were washed with PBS and fixed with absolute methanol for 15 min. After staining cells with crystal violet solution for 15 min, the cells were washed with water, then dried and photographed.

### Wound healing assays

Cells were seeds into 12 wells cell culture plate. When the cells filled the dish, run a 10 μL Tips across the bottom of the plant to create a wound and take a photo to record its width. The cells were cultured in FBS-free medium for 24 h and photographed at the same location to record the wound healing.

### Transwell migration and invasion assays

2 × 10^4^ cells were seed into Transwell pore polycarbonate membrane insert (8 μm) to perform cell migration assay and Matrigel (Corning)-coated chambers were used to detected cell invasion. Lower chambers were added with 800 μL complete medium to co-culture 24 h. Adhered to lower surface cells were fixed with 4% paraformaldehyde and stained with crystal violet. The migrated or invaded cells were captured with a microscope.

### Tube formation assays

HUVEC were seeded into 24 wells cell culture plate coated with Matrigel (Corning) and culture with SKOV3 or HEY cells medium for 4 h. Tube formation were observed with microscope.

### Cell apoptosis analysis

Cells were seeded into 6-well plates and treated when the cell density reached 50%. After 48 h culture, the cells were digested and collected into tubes, then stained with PI and FITC-Annexin Ⅴ for 15 mins according to the cell apoptosis kit manufacturer's instructions (Beyotime). Experiments were performed using a MoFlo XDP flow cytometer sorting system (Beckman Coulter).

### Animal studies

In* vivo* experiments were conducted using female BALB/c nude mice (specific pathogen-free; 4 weeks old; weighing approximately 18 ± 2 g). The mice were provided by Jiangsu Jicui Yaokang Biotechnology Co., Ltd. (Animal License Number SCXK 2018-0008, Nanjing, China) and housed in the SPF Animal Laboratory of Shanghai Hospital of Traditional Chinese Medicine (Animal License Number SYXK 2020-0014) prior to the experiment. All experiments were conducted in accordance with the guidelines of the Ethics Committee of Shanghai Hospital of Traditional Chinese Medicine. After one week of acclimation, all nude mice were anesthetized with isoflurane, and a 1 cm longitudinal incision was made from the dorsal midline of the back to the upper edge of the iliac crest. The peritoneal cavity was opened, and 10 μl of cell suspension containing a total of 5×10^5^ cells was injected deep into the left ovary. The ovary was fixed and compressed for 1 minute, followed by closure of the peritoneum with 5-0 absorbable suture and intermittent 3-0 suture of subcutaneous tissue and skin. The wound was sterilized again.After one week of tumor growth confirmed by fluorescence imaging of small animals, the nude mice were randomly divided into four groups. The PS VII group and the PARPi group received intraperitoneal injections of PS VII and PARPi, respectively. The PS VII+PARPi group received half-dose injections of both drugs, while the NC group received an injection of an equivalent volume of physiological saline. The intervention lasted for a total of 30 days, and the size of the tumor of each group of nude mice was observed again using a fluorescence imaging of small animals system. The mice were then euthanized, and the ovarian cancer tumors were excised, weighed, and subjected to H&E staining and immunohistochemistry. The experimental protocol was approved by the Animal Care and Use Committee of Shanghai Hospital of Traditional Chinese Medicine (approval number 2022025, May 19, 2022).

### Zebrafish assays

We utilized the zebrafish model to microinject PS VII-treated green fluorescent protein (EGFP) labeled resistant HEY cells and their control cells into the yolk sac of zebrafish embryos. The embryos were allowed to develop for 24 hours. After 36 hours of culture, the yolk sacs were observed to assess the migration and dissemination of ovarian cancer cells in the zebrafish embryos. PS VII interventions were performed in zebrafish to observe the effects of PS VII on the development of zebrafish embryos.

### Statistical analysis

Each experiment was performed in triplicate. qRT-PCR data were analyzed by Student's t-test (GraphPad Prism 8). The means ± SEM of three or more independent experiments are reported. Values of P ≤ 0.05 were considered statistically significant.

## Results

### Glycolysis and angiogenesis drive PARP inhibitor resistance in ovarian cancer cells

**Figure 1:**
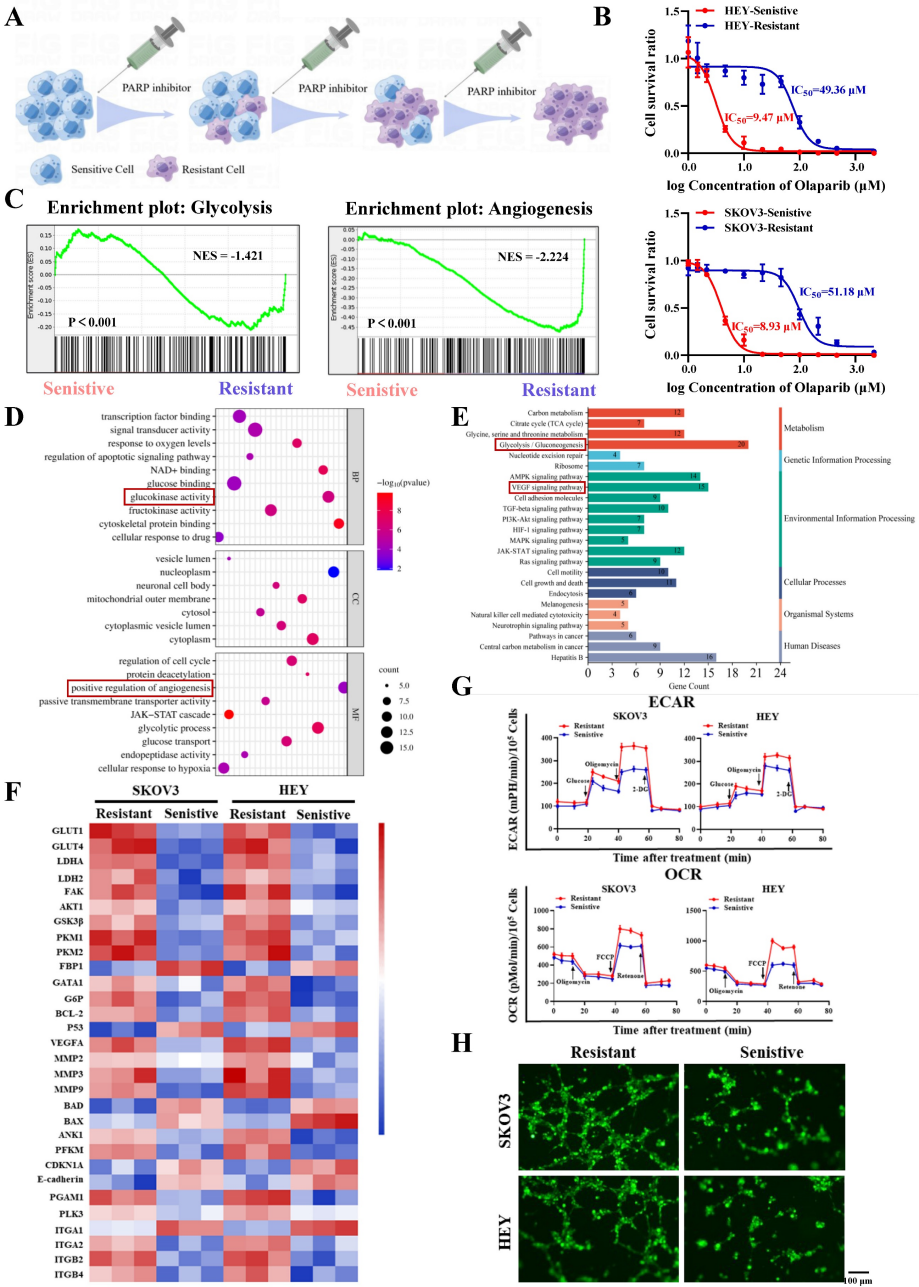
** Increased glycolysis and angiogenesis in ovarian cancer PARP inhibitor-resistant cells. A.** Ovarian cancer PARP inhibitor-resistant cells were established by increasing concentrations of PARP inhibitors; **B.** The half-maximal inhibitory concentration (IC_50_) of PARP inhibitors on the viability of ovarian cancer PARP inhibitor-resistant cells and sensitive cells was determined using CCK-8 assay; **C-F.** RNA-Seq was performed to analyze the gene expression profile of ovarian cancer PARP inhibitor-resistant cells and sensitive cells, showing significant changes in gene expression. GSEA analysis revealed different functional categories **(C).** GO analysis identified different biological functions and molecular mechanisms **(D).** KEGG analysis revealed different signaling pathways **(E).** Heatmaps showed differential gene changes involved in glycolysis, angiogenesis, and oxidative phosphorylation processes** (F)**; **G.** Extracellular acidification rate (ECAR) curves of ovarian cancer cells were monitored. Metabolic inhibitors were injected at different time points. Oligomycin, FCCP, or rotenone was used to measure oxygen consumption rate (OCR) curves of ovarian cancer cells; **H.** The impact of ovarian cancer PARP inhibitor-resistant cells and sensitive cells on the tube formation ability of human umbilical vein endothelial cells (HUVECs). Cell culture supernatants from ovarian cancer PARP inhibitor-resistant cells and sensitive cells were used. The scale bar in 40× images represents 100 μm.

Firstly, we constructed PARP inhibitor-resistant SKOV3 and HEY cell lines by exposing them to increasing concentrations of PARP inhibitor (olaparib) and maintaining the drug pressure for 6 months (Figure 1A). Subsequently, CCK8 assays showed that the half-maximal inhibitory concentration (IC_50_) values of olaparib-treated SKOV3/Resistant and HEY/Resistant cells were 51.18µM and 49.36µM, respectively, while the corresponding parental SKOV3/Sensitive and HEY/Sensitive cells had IC_50_ values of 8.93µM and 9.47µM, confirming the successful construction of PARP inhibitor-resistant SKOV3 and HEY cell lines (Figure 1B). To determine the biological functions driving PARP inhibitor resistance, we utilized RNA-seq to compare the differential gene expression between PARP inhibitor-resistant and sensitive cells. Surprisingly, we found that the differentially expressed genes in both cell types were enriched in biological functions related to glycolysis and angiogenesis (Figure 1C-E). Moreover, many important regulatory genes related to glycolysis and angiogenesis were enriched in PARP inhibitor-resistant cells (Figure 1F). To validate the conclusions of RNA-seq, we tested whether there were differences in glycolysis and angiogenesis functions between PARP inhibitor-resistant and sensitive cell lines. We used a Seahorse XF24 analyzer to monitor the extracellular acidification rate (ECAR) profiles of SKOV3/Resistant, HEY/Resistant, SKOV3/Sensitive, and HEY/Sensitive cells, injecting metabolic inhibitors at different time points over 80 minutes and concurrently measuring the oxygen consumption rate (OCR) of ovarian cancer cells using oligomycin, FCCP, or rotenone. The results demonstrated significantly elevated glucose metabolism levels in PARP inhibitor-resistant cells (Figure 1G). To further confirm the differences in angiogenesis levels between PARP inhibitor-resistant and sensitive cells, we co-cultured human umbilical vein endothelial cells (HUVEC) with SKOV3/Resistant, HEY/Resistant, or their respective control cells for 6 hours. The results showed enhanced angiogenesis in the PARP inhibitor-resistant cells (Figure 1H). Based on these findings, we speculate that glycolysis and angiogenesis likely play important regulatory roles in the process of PARP inhibitor resistance.

### The natural compound PS VII can effectively inhibit the proliferation, migration, and anti-apoptotic ability of PARP inhibitor-resistant cells

**Figure 2:**
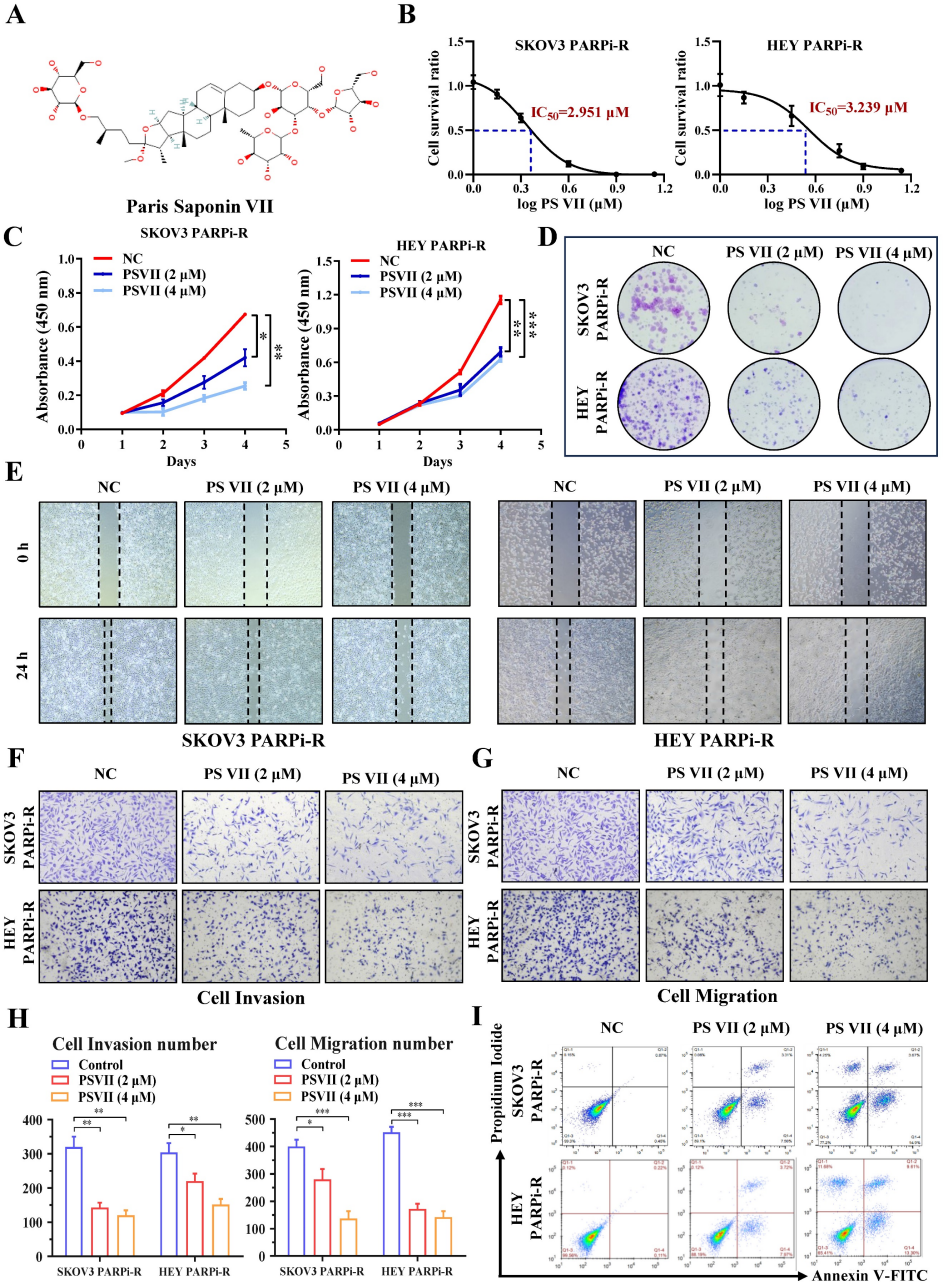
** PS VII inhibits proliferation, metastasis, and anti-apoptosis of ovarian cancer PARP inhibitor-resistant cells. A.** Chemical structure of PS VII; **B.** The half-maximal inhibitory concentration (IC_50_) of PS VII on the viability of ovarian cancer SKOV3 PARPi-R and HEY PARPi-R cells was determined using CCK-8 assay; **C-D.** The effects of different concentrations of PS VII on the proliferation rate of ovarian cancer SKOV3 PARPi-R and HEY PARPi-R cells were detected by CCK-8 **(C)** and colony formation assays **(D)**; **E-H.** The effects of different concentrations of PS VII on the migration and invasion abilities of ovarian cancer SKOV3 PARPi-R and HEY PARPi-R cells were evaluated by wound healing assays **(E)** and Transwell assays **(F-H)**; **I.** The apoptosis of ovarian cancer SKOV3 PARPi-R and HEY PARPi-R cells was determined using flow cytometry at different concentrations of PS VII. *P < 0.05, **P < 0.01, ***P < 0.001.

Natural compounds are considered to be biologically friendly and have shown promising therapeutic effects. In recent years, numerous studies have shown that natural compounds can to some extent reverse tumor resistance. PS VII, derived from the dried rhizomes of plants in the Liliaceae family, such as Paris polyphylla var. yunnanensis or Paris polyphylla var. chinensis, has been used for anti-tumor treatment in Asian countries for thousands of years (Figure 2A). Our previous research has also indicated its ability to effectively inhibit glycolysis in ovarian cancer cells. Based on this, we hypothesized that PS VII might also play a role in inhibiting ovarian cancer PARP inhibitor resistance through glycolysis inhibition. To confirm this hypothesis, we performed CCK8 assays and found that PS VII had significant anti-tumor activity against SKOV3 PARPi-R and HEY PARPi-R cells, with half-maximal inhibitory concentration (IC_50_) values of 2.951 µM and 3.239 µM, respectively (Figure 2B). Additionally, we observed that PS VII could inhibit the proliferation of ovarian cancer SKOV3 PARPi-R and HEY PARPi-R cells (Figure 2C). Colony formation assays further confirmed the inhibitory effect of PS VII on ovarian cancer SKOV3 PARPi-R and HEY PARPi-R cells (Figure 2D). As angiogenesis and resistance are closely associated with tumor metastasis, we investigated the impact of PS VII on the migration and invasion phenotype of ovarian cancer SKOV3 PARPi-R and HEY PARPi-R cells.

The experiments demonstrated that PS VII effectively inhibited the migration and invasion abilities of ovarian cancer SKOV3 PARPi-R and HEY PARPi-R cells (Figure 2E-H). Finally, flow cytometry experiments revealed that PS VII significantly promoted apoptosis in SKOV3 PARPi-R and HEY PARPi-R cells (Figure 2I). These preliminary findings suggest that PS VII may have a potent inhibitory effect on ovarian cancer PARP inhibitor-resistant cells, possibly contributing to the reversal of PARP inhibitor resistance in ovarian cancer.

### PS VII inhibits glycolysis and angiogenesis in PARP inhibitor-resistant cells and exhibits synergistic therapeutic effects with PARP inhibitors

**Figure 3:**
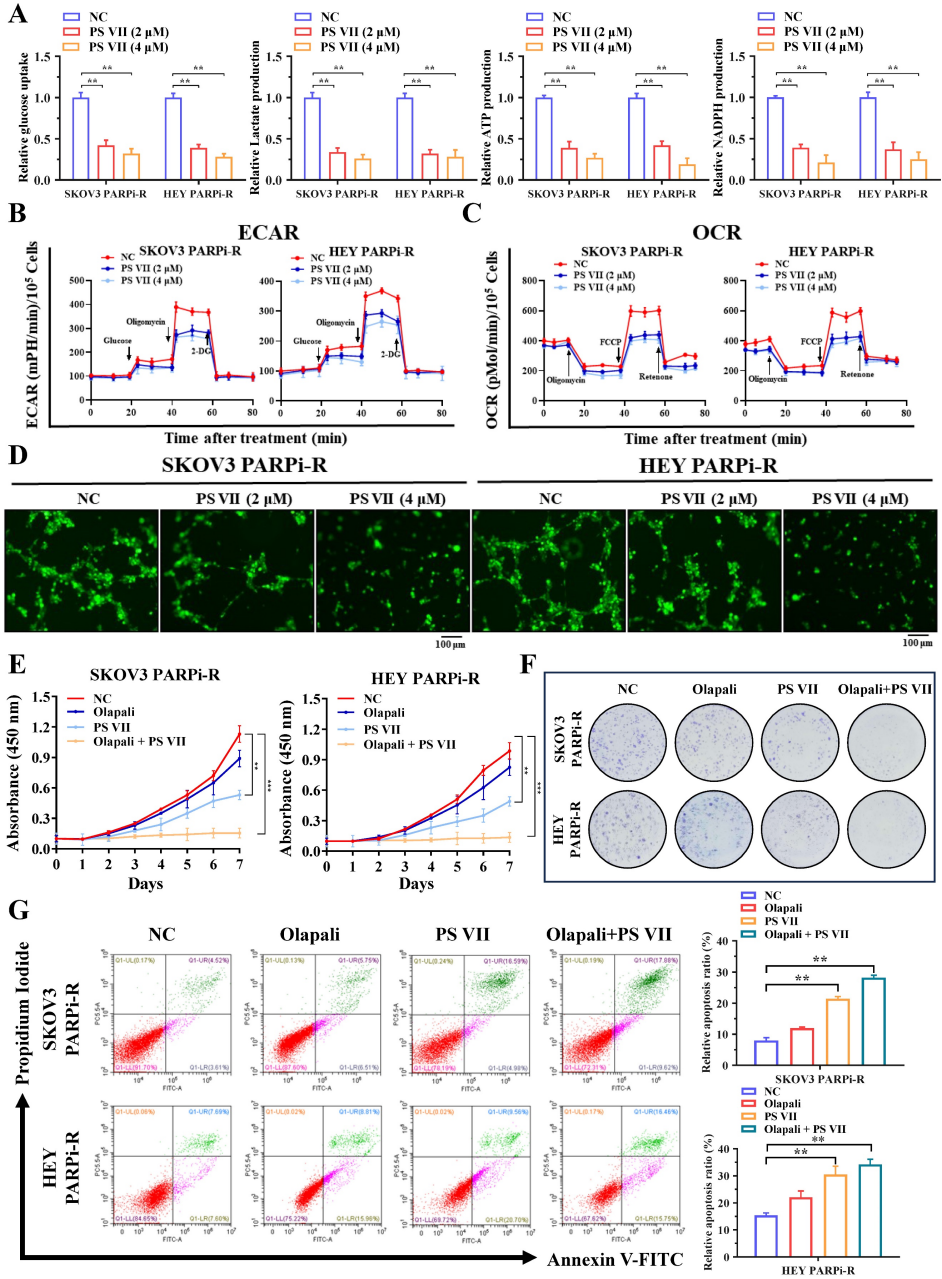
** PS VII inhibits glycolysis and angiogenesis in PARP inhibitor-resistant cells and synergizes with PARP inhibitors for therapeutic effect. A.** Glucose uptake, lactate production, adenosine triphosphate (ATP) generation, and nicotinamide adenine dinucleotide phosphate (NADPH) generation in ovarian cancer SKOV3 PARPi-R and HEY PARPi-R cells were measured after PS VII intervention at different concentrations; **B.** The extracellular acidification rate (ECAR) curves of ovarian cancer SKOV3 PARPi-R and HEY PARPi-R cells after PS VII intervention were monitored. Metabolic inhibitors were injected at different time points; **C.** The oxygen consumption rate (OCR) curves of ovarian cancer SKOV3 PARPi-R and HEY PARPi-R cells after PS VII intervention were measured using oligomycin, FCCP, or rotenone; **D.** The impact of ovarian cancer SKOV3 PARPi-R and HEY PARPi-R cells with different concentrations of PS VII on the tube formation ability of human umbilical vein endothelial cells (HUVECs). Cell culture supernatants from ovarian cancer PARP inhibitor-resistant cells after PS VII intervention were used. The scale bar in 40× images represents 100 μm; **E-F.** The effects of PS VII and PARP inhibitors alone or in combination on the proliferation rate of ovarian cancer SKOV3 PARPi-R and HEY PARPi-R cells were evaluated by CCK-8 **(E)** and colony formation assays **(F)**; **G.** The apoptosis of ovarian cancer SKOV3 PARPi-R and HEY PARPi-R cells treated with PS VII and PARP inhibitors alone or in combination was determined using flow cytometry. *P < 0.05, **P < 0.01, ***P < 0.001.

Our previous data indicated that glycolysis and angiogenesis may play important regulatory roles in the process of PARP inhibitor resistance. To further clarify the role of PS VII in treating ovarian cancer PARP inhibitor resistance, we examined the effects of PS VII intervention on the glycolysis and angiogenesis levels of ovarian cancer PARP inhibitor-resistant cells. Using glucose uptake, lactate production, NADPH generation, ATP generation, and OCR/ECAR detection, we found that PS VII effectively inhibited the glycolysis level of ovarian cancer SKOV3 PARPi-R and HEY PARPi-R cells (Figure 3A-C). In co-culture experiments with HUVECs, PS VII also showed a significant inhibitory effect on tube formation in Matrigel (Figure 3D). Moreover, to investigate whether PS VII could reverse PARP inhibitor resistance in ovarian cancer, we treated SKOV3 PARPi-R and HEY PARPi-R cells with PS VII, PARP inhibitor (olaparib), or their combination and tested their proliferation using CCK8 assays. The results showed that the combination of PS VII and olaparib significantly inhibited the proliferation of SKOV3 PARPi-R and HEY PARPi-R cells (Figure 3E). Colony formation assays also indicated that the combination treatment of PS VII and olaparib was more effective in inhibiting SKOV3 PARPi-R and HEY PARPi-R cells compared to their individual treatments (Figure 3F). Finally, flow cytometry experiments revealed that the combination of PS VII and olaparib significantly promoted apoptosis in SKOV3 PARPi-R and HEY PARPi-R cells (Figure 3G). Based on the above experimental results, we found that PS VII could to some extent reverse the resistance of SKOV3/Resistant and HEY/Resistant cells to PARP inhibitors.

### PS VII directly binds to RORα and stabilizes RORα protein expression

**Figure 4:**
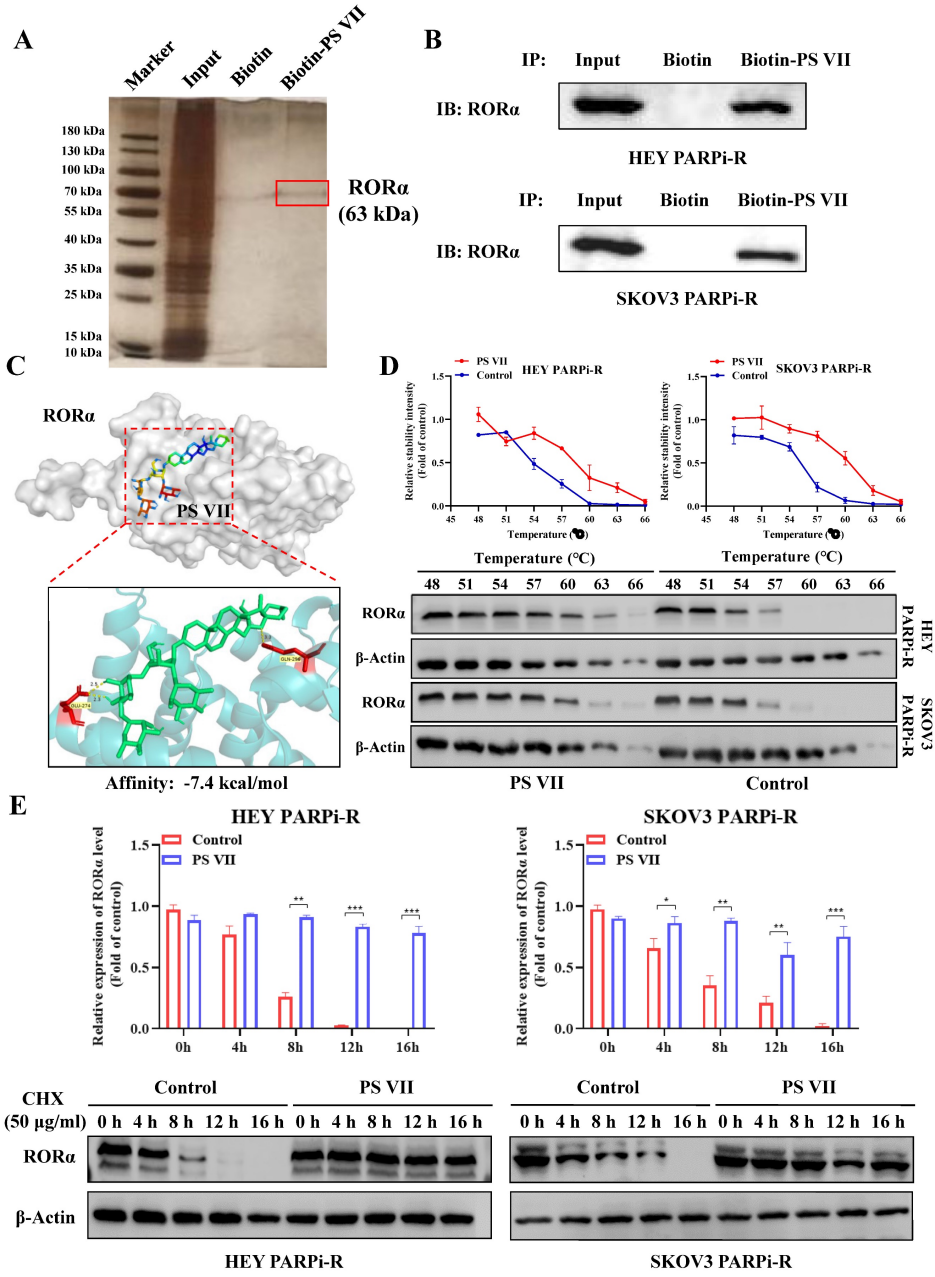
** Direct Interaction of PS VII Stabilizes RORα Protein Expression. A, B.** co-immunoprecipitation (IP) experiments and Mass spectrometry analysis using biotin-labeled PS VII confirm the interaction between PS VII and RORα; **C.** Molecular docking analysis identifies potential binding regions between PS VII and RORα; **D.** Cellular thermal shift assay (CETSA) demonstrates that PSVII can inhibit RORα protein degradation; **E.** Immunoblot analysis shows the stability of RORα protein after CHX treatment at different time points. *p < 0.05, **p < 0.01, ***p < 0.001.

To explore the in-depth regulatory mechanism of PS VII, we used biotinylated PS VII for immunoprecipitation (IP) analysis to identify potential protein targets that could bind to PS VII. A total of 66 proteins potentially interacting with PS VII were identified through SDS-PAGE silver staining and mass spectrometry analysis, among which RORα is one of the potential binding proteins for PS VII (Figure 4A). In our previous studies, we have found that PS VII binding stabilizes the protein stability not only of RORα but also of the RORC protein, another member of the ROR family. In this study, we similarly observed that PS VII can bind to RORα and further enhance the protein stability of RORα, which may contribute to the regulation of its biological functions. Subsequently, we performed Co-IP-western blot analysis to validate the binding interaction between PS VII and RORα. The results showed that PS VII has a certain binding ability to RORα (Figure 4B). In order to further identify the direct binding between PS VII and RORα, molecular docking experiments targeting PS VII and RORα also indicated a favorable binding domain between them (Figure 4C). Moreover, cellular thermal shift assay (CETSA) experiments based on the biophysical principle of ligand-induced thermal stabilization of potential target proteins were conducted. The results showed that as the temperature gradually increased from 48°C to 66°C, PS VII intervention significantly increased the protein expression of RORα compared to the control group, indicating a direct interaction between PS VII and RORα through affecting thermal stability (Figure 4D). Furthermore, we used cycloheximide (CHX) to inhibit protein synthesis and further verified the effect of PS VII on RORα protein degradation. The results demonstrated that binding of PS VII significantly stabilized the protein level of RORα and increased the half-life of RORα protein (Figure 4E).

### PS VII regulates glycolysis and angiogenesis characteristics in PARP inhibitor-resistant cells by modulating RORα

**Figure 5:**
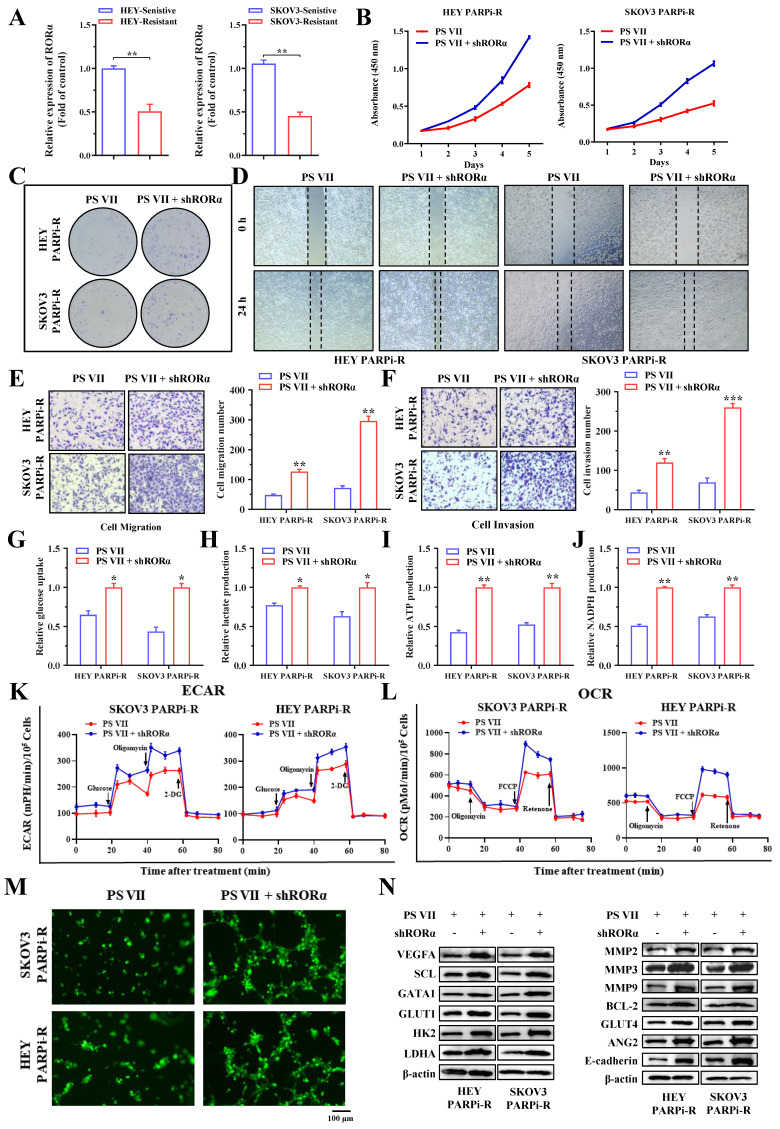
** PS VII Regulates RORα and Impacts Glycolysis and Angiogenesis in PARPi-Resistant Cells. A.** qRT-PCR is used to examine the expression of RORα mRNAs in PARP inhibitor-resistant and sensitive cell lines; **B-C.** CCK-8 assay **(B)** and colony formation assay **(C)** are used to evaluate the effect of RORα inhibition on the proliferative rate of PARPi-resistant ovarian cancer cells treated with PS VII; **D-F.** Wound healing assay **(D)** and Transwell migration and invasion assays **(E-F)** are employed to determine the impact of RORα inhibition on the migratory and invasive abilities of PARPi-resistant ovarian cancer cells treated with PS VII; **G-J.** RORα inhibition is assessed for its effect on glucose uptake **(G)**, lactate production **(H)**, ATP production **(I)**, and NADPH production **(J)** in PARPi-resistant ovarian cancer cells treated with PS VII; **K.** Extracellular acidification rate (ECAR) curves are monitored to evaluate the effect of RORα inhibition on glycolysis in PARPi-resistant ovarian cancer cells treated with PS VII at different time points upon injection of metabolic inhibitors; **L.** Oligomycin, FCCP, or rotenone is used to assess the effect of RORα inhibition on the oxygen consumption rate (OCR) curves in PARPi-resistant ovarian cancer cells treated with PS VII; **M.** The impact of PS VII and RORα on tube formation in human umbilical vein endothelial cells (HUVECs) is examined using conditioned medium derived from PARPi-resistant ovarian cancer cells with RORα inhibition treated with PS VII. The scale bar in 40× images represents 100 μm; **N.** The effect of PS VII and RORα on glycolysis, angiogenesis, and metastasis-related proteins in PARPi-resistant ovarian cancer cells is analyzed using Western blot. *p < 0.05, **p < 0.01, *** p < 0.001.

To validate the function of RORα in the regulation of PARP inhibitor-resistant cells by PS VII, we first examined the expression of RORA in PARP inhibitor-resistant and sensitive cell lines, and the results showed that RORA was significantly decreased in PARP inhibitor-resistant cells (Figure 5A). Subsequently, we downregulated the expression of RORα in SKOV3 PARPi-R and HEY PARPi-R cells using shRNA. We confirmed our hypothesis through CCK8 assays and colony formation assays, showing that the downregulation of RORα expression inhibited the therapeutic effects of PS VII (Figure 5B-C).

Next, we also examined the impact of PS VII on the migration and invasion phenotypes of ovarian cancer cells after downregulating RORα expression. The experimental results showed that the inhibitory ability of PS VII towards the migration and invasion ability of ovarian cancer SKOV3 PARPi-R and HEY PARPi-R cells was similarly diminished after downregulating RORα expression (Figure 5D-F). We further investigated whether RORα regulates glycolysis and angiogenesis levels in ovarian cancer cells. We examined glucose uptake, lactate production, NADPH generation, ATP generation, OCR, and ECAR levels in cells with downregulated RORα expression. We found that the inhibitory effect of PS VII on the glycolysis level of ovarian cancer SKOV3 PARPi-R and HEY PARPi-R cells was reversed when RORα expression was downregulated (Figure 5G-L). In co-culture experiments with HUVECs, PS VII intervention in SKOV3 PARPi-R, HEY PARPi-R cells, and their corresponding RORα shRNA cells demonstrated that downregulating RORα expression hindered the inhibitory effect of PS VII on tube formation in Matrigel (Figure 5M). To explore the potential underlying mechanism of RORα-mediated glycolysis and angiogenesis, we subsequently used Western blot to detect the levels of key proteins associated with these functions. We found that inhibiting RORα expression in PS VII-treated SKOV3 PARPi-R, HEY PARPi-R cells altered the levels of proteins related to cell glycolysis, angiogenesis, and metastasis (Figure 5N). These results suggest that PS VII may modulate glycolysis and angiogenesis characteristics in ovarian cancer PARP inhibitor-resistant cells by regulating RORα.

### ECM1 is a direct target of RORα in ovarian cancer cells

**Figure 6:**
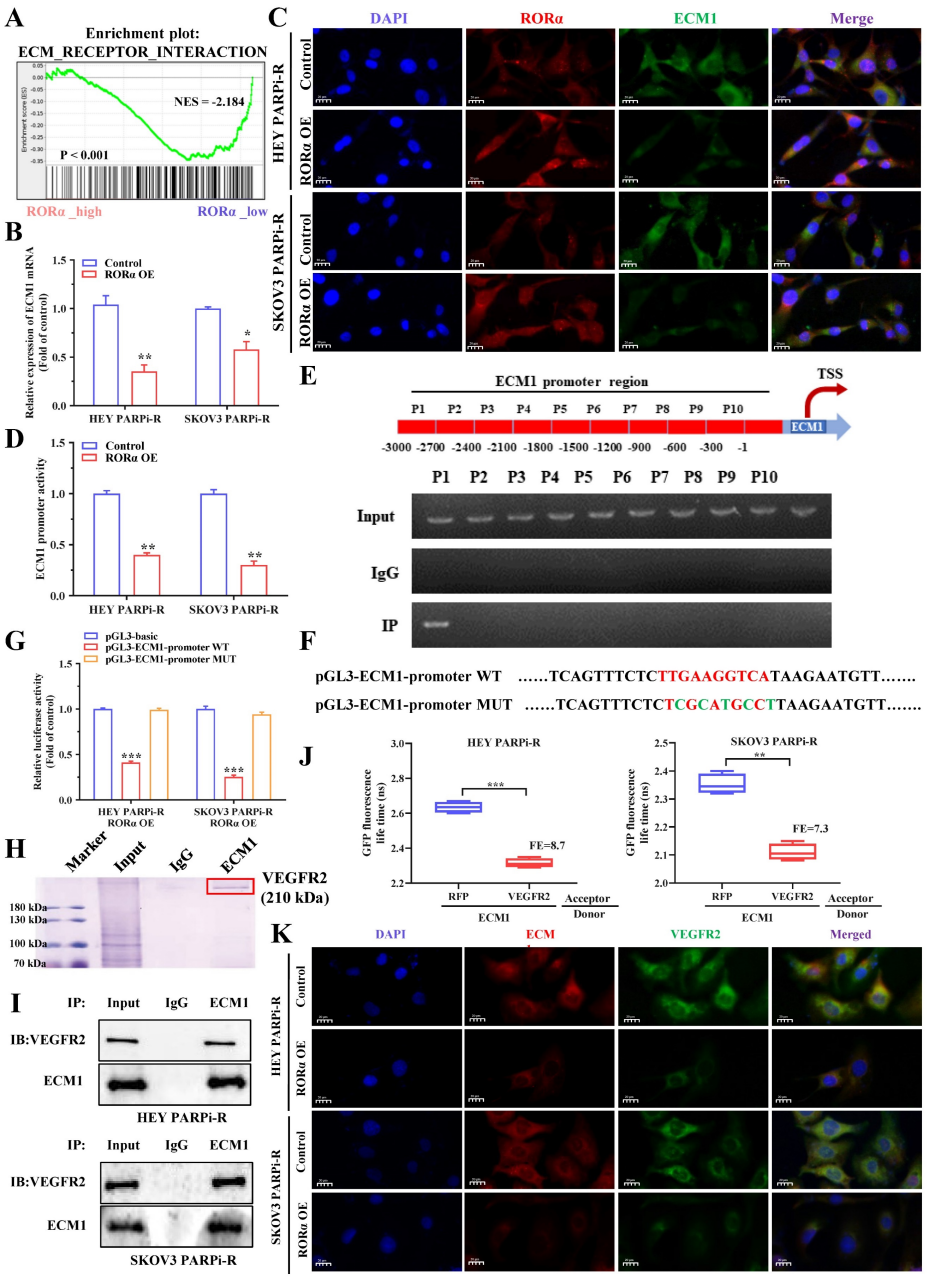
** RORα Modulates ECM1/VEGFR2 Signaling Pathway. A.** GSEA analysis is performed using RORα overexpressing ovarian cancer cells and control cells; **B.** qRT-PCR is used to measure the mRNA levels of ECM1 in RORα overexpressing and control ovarian cancer cells; **C.** Immunofluorescence staining (IF) demonstrates the relationship between RORα and ECM1; **D.** Luciferase activity assay is conducted to assess the activity of ECM1 promoter in HEY/RORα OE and SKOV3/RORα OE cells; **E.** ChIP-qRT-PCR shows the binding of RORα to the promoter region of ECM1 gene; **F-G.** Luciferase reporter gene assay is performed to investigate the binding sites of RORα in the promoter region of ECM1; **H.** IP assays and mass spectrometry of stained proteins showed that VEGFR2 binds to ECM1; **I.** Co-IP experiments confirm that ECM1 and VEGFR2 can directly bind; **J.** The interaction between ECM1 and VEGFR2 is validated using FRET-FLIM technology in a transient co-expression system. FE represents FRET efficiency; **K.** Immunofluorescence staining (IF) further confirms the relationship between ECM1 and VEGFR2. *p < 0.05, **p < 0.01, *** p < 0.001.

The above results indicate that PS VII can downregulate glycolysis and angiogenesis in ovarian cancer PARP inhibitor-resistant cells through the RORα signaling pathway. To understand the signaling pathway of RORα in ovarian cancer cells, we performed RNA-seq analysis on cells with knocked-down or overexpressed RORα in PARP inhibitor-resistant cells. The results showed a close correlation with the ECM1 protein family (Figure 6A). Therefore, we verified through PCR experiments that ECM1 is negatively correlated with RORα (Figure 6B). Immunofluorescence double staining experiments of RORα and ECM1 in HEY and SKOV3 cells confirmed a negative correlation between their expression levels (Figure 6C). As RORα is known to be a repressive transcription factor, we hypothesized that RORα may impact the transcriptional expression of ECM1. We subsequently used a luciferase reporter gene assay to confirm that RORα can reduce the luciferase activity of the ECM1 promoter (Figure 6D). To confirm the RORα binding region in the ECM1 promoter, we performed ChIP assays and found that a binding site was located approximately 2,700 bp upstream of the transcription start site in a RORα binding region (Figure 6E). We then performed luciferase reporter gene assays to confirm the mechanism between RORα and ECM1. Wild-type (WT) ECM1 promoter plasmids (pGL3-ECM1-promoter WT) and mutant-type ECM1 promoter plasmids (pGL3-ECM1-promoter MUT) were transfected into HEY/RORα OE and SKOV3/RORα OE cells. The results showed that RORα inhibited the luciferase activity of the ECM1 promoter, and the mutation at the binding site restored the luciferase activity (Figure 6F-G). These results indicate that RORα can act as a transcription factor to inhibit ECM1 expression.

### PS VII reverses PARP inhibitor resistance in ovarian cancer cells through the RORα/ECM1/VEGFR2 signaling pathway

To further verify the targeted regulatory relationship between RORα and ECM1 under PS VII intervention, we used shRNA to knock down ECM1 expression in SKOV3/RORα KD cells (Supplementary Figure 1A) and conducted a rescue experiment on the therapeutic efficacy of PS VII.

We found that the expression inhibition of ECM1 partially reversed the effect of RORα downregulation on PS VII therapy (Supplementary Figure 1B-C). The inhibition of ECM1 expression was also observed to rescue cell migration and angiogenesis (Supplementary Figure 1D-E) and to restore the glucose uptake and lactate, ATP, and NADPH efficacy of PS VII against PARP inhibitor-resistant ovarian cancer cells under RORα downregulation (Supplementary Figure 1F). In addition, downregulation of ECM1 in ovarian cancer cells also restored the impact of PS VII on ECAR and OCR (Supplementary Figure 1G). At the protein level, the expression downregulation of ECM1 and RORα restored the expression levels of the VEGFA, GLUTI, HK2, GLUT2, and SCL proteins after PS VII intervention (Supplementary Figure 1H). Therefore, we believe that the RORα/ECM1 axis plays an important role in PS VII's treatment of PARP inhibitor-resistant ovarian cancer. To further explore the regulatory mechanism of ECM1, we performed IP assays to analyze the proteins that bind to ECM1, mass spectrometry of stained proteins showed that VEGFR2 was a potential binding protein of ECM1 (Figure 6H).

We subsequently confirmed the interaction between ECM1 and VEGFR2 through Co-IP and FRET-FLIM experiments (Figure 6I-J). Immunofluorescence double staining experiments on ECM1 and VEGFR2 in HEY PARPi-R and SKOV3 PARPi-R cells also confirmed the correlation between the expression of ECM1 and VEGFR2 (Figure 6K). To verify the role of VEGFR2, we performed a rescue experiment on VEGFR2, and used shRNA to knock down the expression of VEGFR2 in HEY/ECM1 OE cells (Supplementary Figure 2A), and again conducted a rescue experiment on the therapeutic efficacy of PS VII. The results also showed that the expression inhibition of VEGFR2 partially reversed the impact of ECM1 overexpression on PS VII's intervention on ovarian cancer cell proliferation, angiogenesis, glycolysis, and other functions (Supplementary Figure 2B-F). Previous studies have confirmed that VEFGR2 can regulate downstream FAK signaling pathways, and to further verify whether ECM1 regulates downstream signaling pathways by binding to VEFGR2, we overexpressed ECM1 in RORαOE cell lines in a rescue experiment and observed the effects on protein expression levels in the signaling pathway. The results showed that ECM1 can rescue the expression levels of p-FAK, p-AKT, and p-GSK3β in PARP inhibitor-resistant ovarian cancer cells (Supplementary Figure 2G). We then examined the expression levels of key molecules in the RORα/ECM1/VEGFR2 signaling pathway in PARP inhibitor-resistant ovarian cancer cells before and after PS VII intervention, and found that PS VII significantly inhibited the expression levels of metabolism and angiogenesis-related molecules such as VEGFR2, SCL, HK2, LDHA, and FAK/AKT1/GSK3β by binding and activating RORα and regulating the downstream ECM1/VEGFR2 axis (Supplementary Figure 2H). Therefore, we conclude that PS VII may hinder the binding of ECM1 to VEGFR2 and inhibit the FAK/AKT/GSK3β signaling pathway in PARP inhibitor-resistant ovarian cancer cells by activating RORα, affecting functions such as glycolysis and angiogenesis, and ultimately improving its resistance treatment.

### PS VII exhibits a synergistic therapeutic effect with PARPi *in vivo* through the RORα/ECM1/VEGFR2 signaling pathway

**Figure 7:**
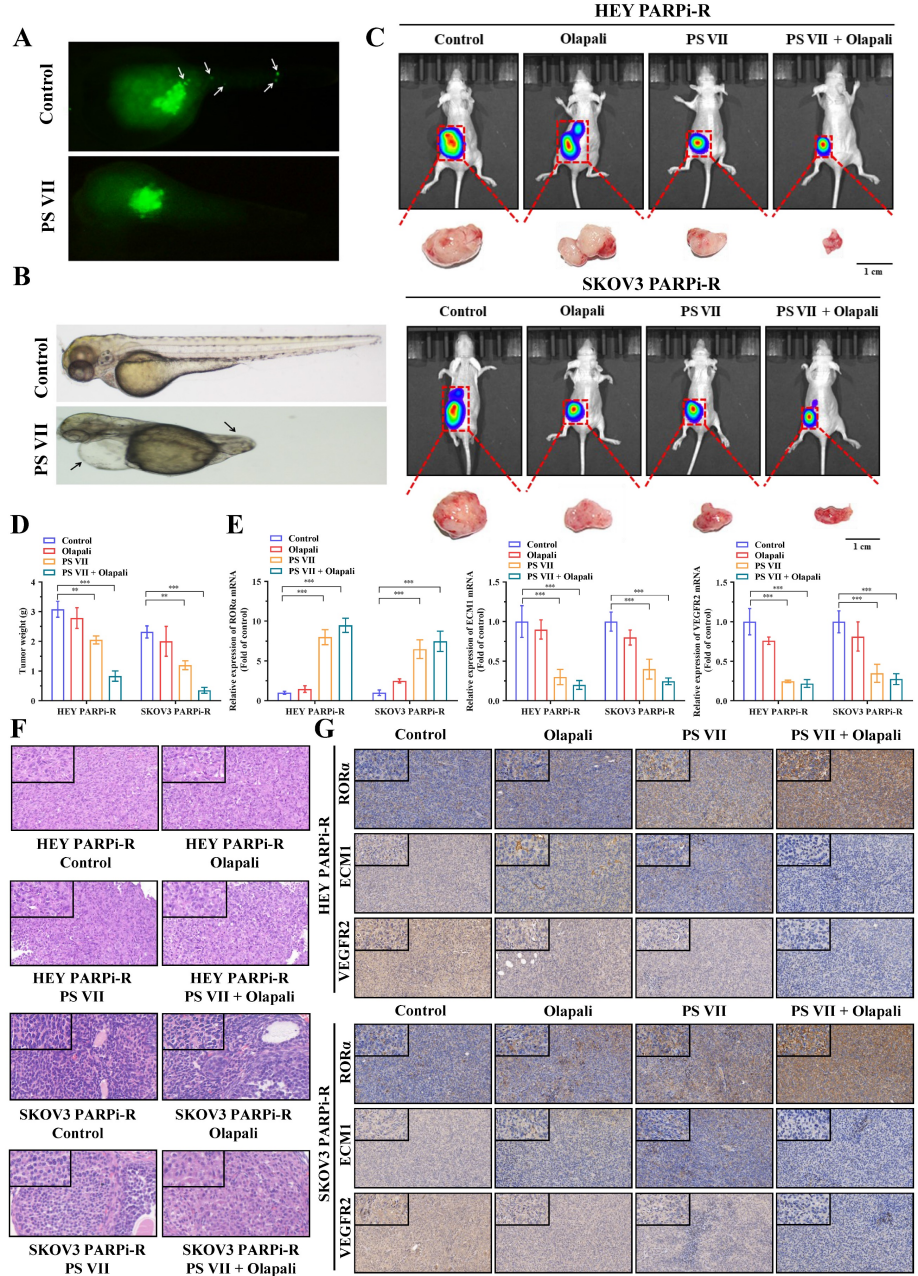
** PS VII and PARPi Synergistically Inhibit Tumor Growth *In Vivo*. A, B.** Zebrafish metastasis model demonstrates the ability of PS VII to inhibit angiogenesis and metastasis of ovarian cancer cells (A, white arrows indicate distant metastasis); **C.** Ovarian orthotopic tumor xenograft models in mice are established using SKOV3 PARPi-R and HEY PARPi-R cells. *In vivo* imaging and tumor images after dissection are obtained following individual or combined intervention with PS VII and PARPi; **D.** Tumor weights are compared among different groups of mice; **E.** qRT-PCR is used to examine the expression of RORα, ECM1, and VEGFR2 mRNAs in the tumor tissues of each group; **F.** H&E staining of tumor tissues is compared among different groups; **G.** Immunohistochemistry (IHC) is performed to evaluate the protein levels of RORα, ECM1, and VEGFR2 in the tumor tissues of each group (all at 100× and 400× magnification); *p < 0.05, **p < 0.01, ***p < 0.001.

To clarify the therapeutic effect of PS VII *in vivo*, we first tested the effect of PS VII on the migration and angiogenesis of PARP inhibitor-resistant ovarian cancer cells using a zebrafish model. We labeled PS VII-intervened PARP inhibitor-resistant ovarian cancer cells with enhanced green fluorescent protein (EGFP) and their corresponding control cells and injected them into the yolk sac of zebrafish embryos. Cell migration and amplification could be observed after 24 hours. After 36 hours of incubation, we found that PS VII intervention effectively inhibited the migration and proliferation of zebrafish embryonic ovarian cancer cells (Figure 7A). Furthermore, through PS VII intervention in zebrafish, we found that after 48 hours of embryonic development, the heart of PS VII-intervened zebrafish expanded significantly, the tail was insufficiently developed, and there was a lack of tissues such as blood vessels and muscles, leading to the development of malformed embryos (Figure 7B).

To simulate the anatomy, blood supply, and tumor microenvironment of human ovarian cancer to the greatest extent possible, we established an *in situ* ovarian cancer nude mouse model with a microenvironment similar to that of the primary tumor by injecting luciferase-carrying SKOV3 PARPi-R and HEY PARPi-R cells into the ovarian tissue. After confirming tumor growth through fluorescence imaging of small animals, we randomly divided the mice into groups and intervened with PS VII and PARPi alone or in combination for a total of 30 days. We then used the fluorescence imaging of small animals system again to observe the tumor size of each group of nude mice, and the results showed that PS VII can exhibit a synergistic therapeutic effect with PARPi, better suppressing ovarian cancer tumor growth (Figure 7C). We then euthanized the mice in each group and isolated the ovarian cancer tumors *in vivo*. We further confirmed the effective inhibition of *in situ* ovarian cancer tumors in nude mice by using PS VII in combination with PARPi through tumor weight measurement (Figure 7D). We tested the changes in the expression of key molecules associated with the RORα/ECM1/VEGFR2 signaling pathway in the bodies of different groups of nude mice after PS VII and PARPi intervention alone or in combination through PCR and immunohistochemical experiments, clearly demonstrating the critical regulatory role of RORα/ECM1/VEGFR2 in the PS VII treatment process (Figure 7E-G). We also examined the expression of the tumor angiogenesis marker CD31, which showed that PS VII in combination with PARPi could significantly inhibit ovarian cancer angiogenesis (Supplementary Figure 3A). Meanwhile, the HE staining of liver and kidney tissues in each group of mice indicated that PS VII, as a natural compound, had good safety (Supplementary Figure 3B). These results collectively demonstrate that PS VII can also exhibit a synergistic therapeutic effect with PARPi *in vivo* through the RORα/ECM1/VEGFR2 signaling pathway and plays a critical role in suppressing the growth and metastasis of PARP inhibitor-resistant ovarian cancer tumors.

### Clinical data confirms the association of RORα/ECM1/VEGFR2 with ovarian cancer PARP inhibitor resistance and prognosis

**Figure 8:**
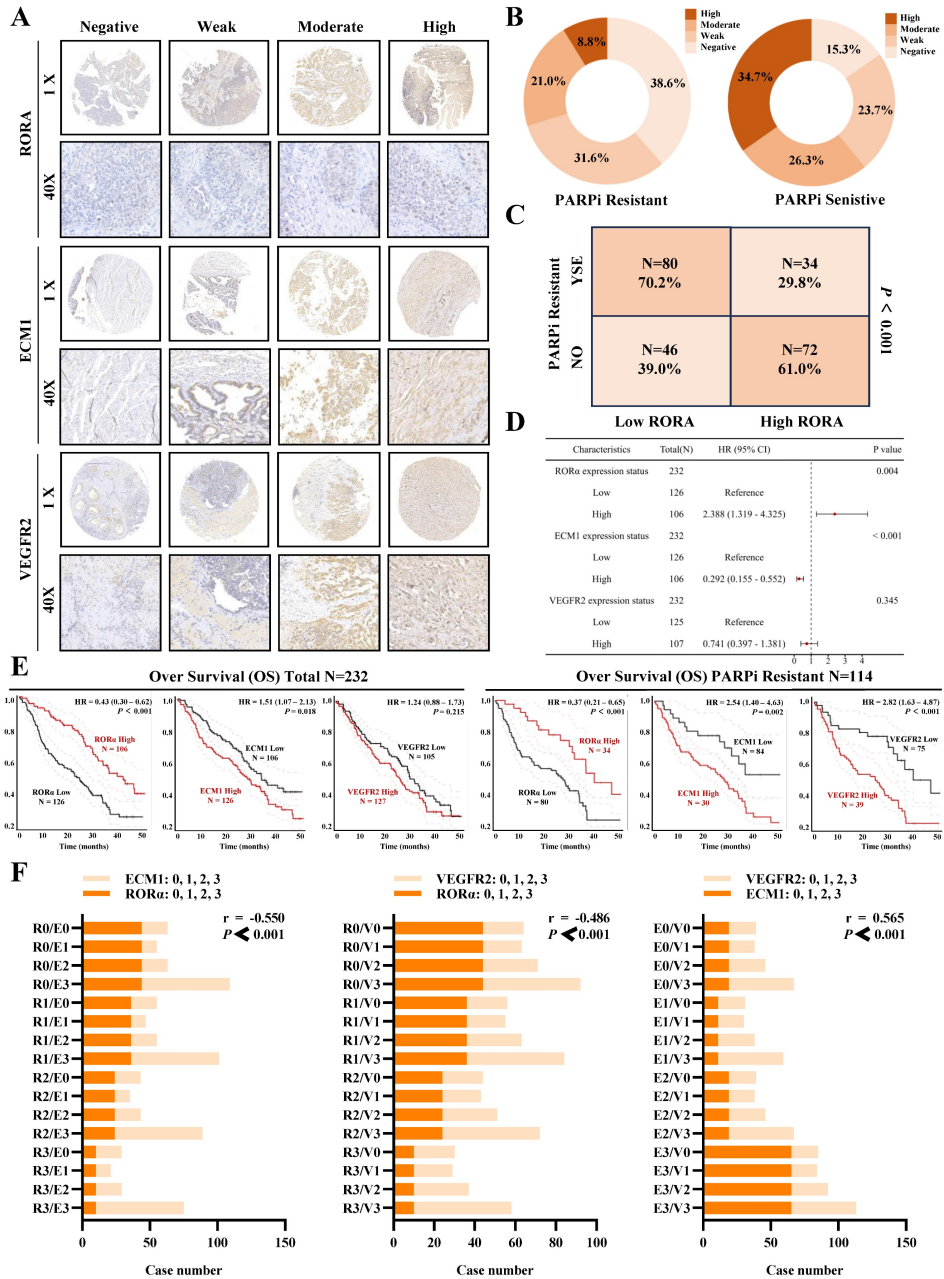
** Clinical Tissue Microarray Suggests the Association of RORα/ECM1/VEGFR2 with PARPi Resistance and Prognosis in Ovarian Cancer. A.** Clinical tissue microarrays containing representative biopsy images showing negative, weak, moderate, and strong expressions of RORα, ECM1, and VEGFR2 (all at 400× magnification); **B-D.** Based on the IHC staining scores, ovarian cancer patients are divided into high and low expression groups for RORα. H represents high; L represents low. The correlation between RORα expression level and PARPi resistance is determined; **E.** Kaplan-Meier analysis is performed to investigate the relationship between RORα, ECM1, VEGFR2, and overall survival in all ovarian cancer patients and PARPi-resistant ovarian cancer patients; **F.** Correlation of IHC staining scores between RORα, ECM1, and VEGFR2 in PARPi-resistant ovarian cancer patients.

**Figure 9:**
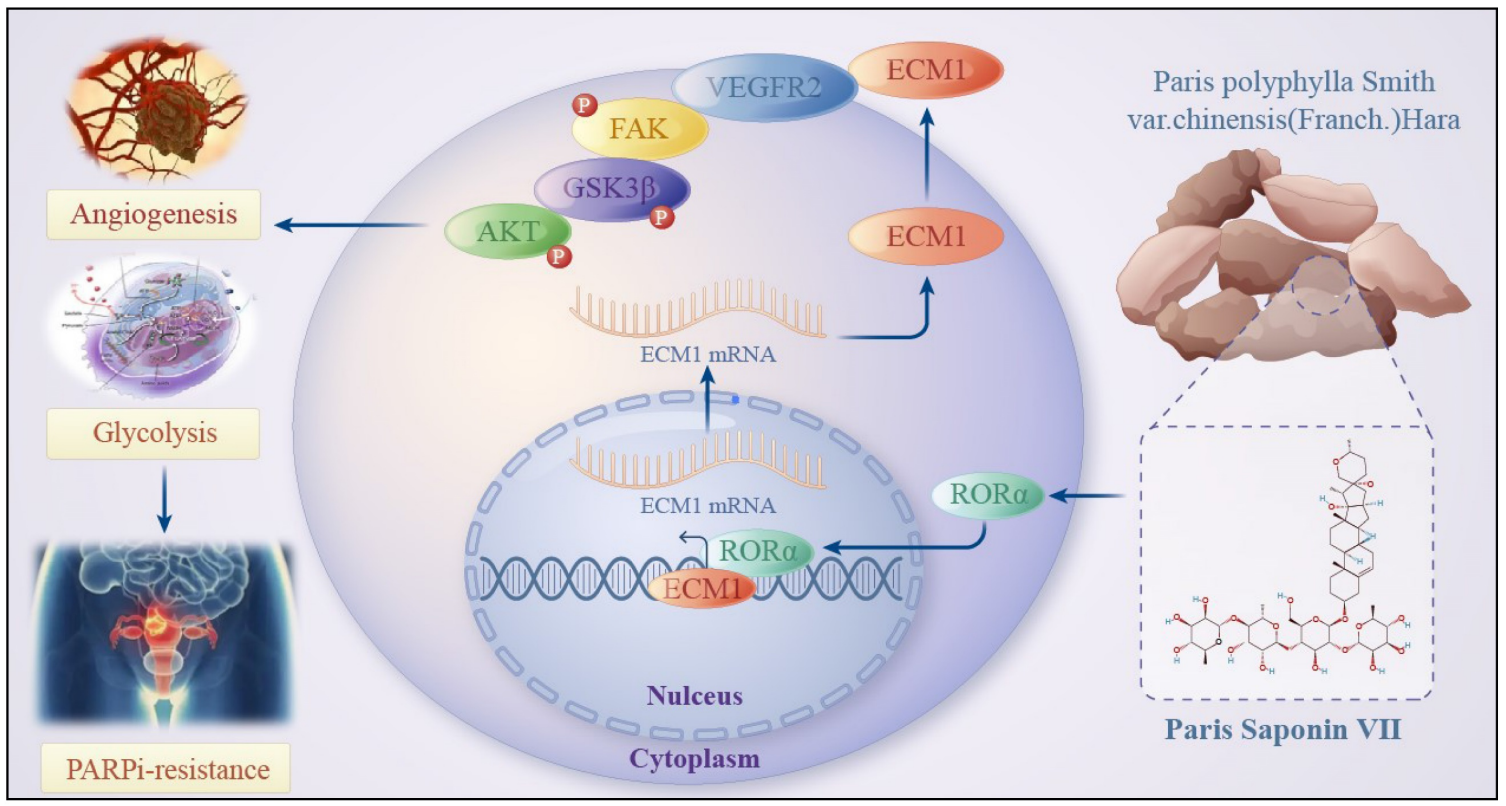
Schematic Diagram of the Mechanism of Rehmannioside VII in Improving PARPi Resistance by Modulating the RORα/ECM1/VEGFR2 Signaling Axis in Ovarian Cancer Tumor Angiogenesis and Glycolysis.

To evaluate the clinical translational application and potential therapeutic efficacy of PS VII, we assessed the expression of key molecules in the RORα/ECM1/VEGFR2 signaling axis, which is the drug target of PS VII, through an ovarian tissue chip. A total of 232 high-grade serous ovarian cancer patient tissue chips (including 114 PARP inhibitor-resistant patients and 118 PARP inhibitor-sensitive patients) were selected to evaluate the clinical significance of RORα, ECM1, and VEGFR2 in ovarian cancer (Figure 8A). The analysis results showed that in the tissue samples of ovarian cancer patients resistant to PARP inhibitors, 8.7% (10/114) showed "High" expression of RORα, 21.0% (24/114) showed "Moderate" expression, 36.6% (36/114) showed "Weak" expression, and 38.6% (44/114) showed "Negative" expression. In the tissue samples of ovarian cancer patients sensitive to PARP inhibitors, 34.7% (41/114) showed "High" expression of RORα, 26.3% (31/114) showed "Moderate" expression, 23.7% (28/114) showed "Weak" expression, and 15.3% (18/114) showed "Negative" expression (Figure 8B). RORα was significantly low expression in ovarian cancer tissue resistant to PARP inhibitors, while it was high expression in sensitive tissue (Figure 8C). In addition, the expression of ECM1 and VEGFR2 in tissue chips was also closely related to PARP inhibitor resistance in ovarian cancer (Supplementary Figure 3A-D). Further analysis of clinical data revealed that low expression of RORα and high expression of ECM1 and VEGFR2 were all associated with poor overall survival (OS) and progression-free survival (PFS) in patients with PARP inhibitor-resistant ovarian cancer. Low expression of RORα and high expression of ECM1 were also closely associated with poor OS and PFS in all ovarian cancer patients (Figure 8D-E, Supplementary Figure 3E, Supplementary Figure 4).The correlation between RORα, ECM1, VEGFR2, and clinical pathological features in ovarian cancer patients is shown in Supplementary Tables 1 and 2. Consistently with our previous observations, the expression of RORα negatively correlated with the expression levels of ECM1 (r = -0.550, *P* < 0.001) and VEGFR2 (r = -0.486, *P* < 0.001), while the expression of ECM1 positively correlated with the expression of VEGFR2 (r = 0.565, *P* < 0.001) (Figure 8F). Based on this, we confirmed once again in clinical samples that the RORα/ECM1/VEGFR2 signaling axis is closely associated with PARP inhibitor resistance, disease progression, and survival prognosis in ovarian cancer. PS VII, a targeted drug for the RORα/ECM1/VEGFR2 signaling axis, is expected to become a new generation of ovarian cancer therapeutic agents (Figure 9).

## Discussion

Ovarian cancer, as the most lethal gynecological malignancy, has the highest mortality rate among female malignancies 21. It often lacks obvious symptoms in the early stages, and even when symptoms are present, they are nonspecific. Most patients are already in the advanced stages at the time of initial diagnosis 22. The ratio of ovarian cancer mortality to incidence exceeds 0.6, and the recurrence rate of advanced ovarian cancer can reach 85%. This poses a significant threat to the life and health of women worldwide, earning it the title of "silent killer". However, in recent years, with the emergence and clinical application of poly(ADP-ribose) polymerase inhibitors (PARPi), the treatment of ovarian cancer has entered a new treatment paradigm 23. It has transitioned from the traditional "surgery+chemotherapy+observation/waiting" model to a comprehensive management model of "surgery+chemotherapy+maintenance therapy" breaking through the treatment bottleneck of ovarian cancer and entering the era of precision medicine 24. Although PARPi is being increasingly used in clinical practice, the development of drug resistance is an urgent clinical challenge that needs to be addressed.

The strategy to overcome tumor drug resistance in modern medicine is similar to antibiotic resistance in the field of infectious diseases. It involves the combination of drugs with non-overlapping mechanisms of action, including substitution combination therapy or the use of new anticancer drugs 25. However, these approaches often have a single target, significant toxic side effects, and can easily lead to secondary or multidrug resistance during the treatment resistance process, further complicating the management of ovarian cancer 26. Traditional Chinese medicine (TCM), with its characteristics of multiple components, targets, and stages of action, has been widely used in clinical cancer treatment in Asian countries 27. In recent years, related studies have found that TCM shows unique effectiveness in enhancing the sensitivity of anticancer drugs and overcoming drug resistance 28.

In this study, we constructed PARP inhibitor-resistant SKOV3 and HEY cell lines and used RNA-seq to compare the differential gene expression between PARP inhibitor-resistant and sensitive cells. We found that glycolysis and angiogenesis play important roles in ovarian cancer PARP inhibitor resistance. Additionally, we confirmed the efficacy of a saponin natural product called VII extracted from the traditional Chinese herb Chonglou in reversing PARP inhibitor resistance in ovarian cancer, showing promising clinical application potential. Mechanistically, we found that VII can bind to RORα, enhance its protein stability, and inhibit the FAK/AKT/GSK3β signaling pathway in ovarian cancer cells resistant to PARP inhibitors through the RORα/ECM1/VEGFR2 signaling axis. This affects ovarian cancer cell functions such as glycolysis and angiogenesis, ultimately reversing the resistance to PARP inhibitors. This innovative drug discovery may become a potential treatment for ovarian cancer PARP inhibitor resistance in the future. Furthermore, the identification of the mechanism of action of this drug provides potential targeted therapeutic targets for ovarian cancer PARP inhibitor resistance.

Paris Saponin VII is an active monomer component of the traditional Chinese herb Chonglou, which comes from the dried rhizome of plants in the lily family, such as Paris polyphylla. Traditional Chinese medicine believes that Chonglou has the effect of clearing heat and detoxifying the body. It has been widely used in clinical cancer treatment in traditional Chinese medicine, with extensive clinical practice showing its cytotoxic effects against tumors 29. Modern pharmacology also suggests that the active monomer PS VII in Chonglou inhibits tumor growth by inhibiting the cell cycle, inducing autophagy, and promoting cell apoptosis. Studies have found that PS VII inhibits the proliferation of lung cancer cells, induces G2/M cell cycle arrest, triggers cell apoptosis, and upregulates p53 expression while inhibiting CCNB1 expression 30. *In vivo* experiments have demonstrated that PS VII can inhibit the growth of xenograft tumors in nude mice, showing good potential in inhibiting tumor progression. PS VII can also downregulate the NF-κB/MMP-9/VEGF signaling pathway, significantly reduce tube formation in human umbilical vein endothelial cells, decrease the number and length of ISV and SIV in transgenic zebrafish 31. Previous studies have shown that PS VII has good anti-tumor effects in various types of cancer. However, current research on PS VII has mainly focused on its effects on cell proliferation and apoptosis, and its anti-cancer mechanism is not fully understood. In our previous research, we have demonstrated that PS VII can inhibit glycolysis, impede the growth of ovarian cancer cells, and promote apoptosis by targeting the RORC/ACK1 signaling pathway 32. This supports the potential development of PS VII as a candidate drug for ovarian cancer. In this study, based on RNA-seq results in PARP inhibitor-resistant SKOV3 and HEY cell lines, we further confirmed that glycolysis and angiogenesis may play important regulatory roles in the process of PARP inhibitor resistance. Moreover, we have discovered the specific mechanism by which PS VII reverses PARP inhibitor resistance in ovarian cancer through the RORα/ECM1/VEGFR2 signaling axis.

Retinoic acid receptor-related orphan receptor alpha (RORα) is a member of the nuclear steroid hormone receptor superfamily. As a member of the retinoic acid-related orphan receptor family, RORα can directly enter the cell nucleus, regulate the transcription of target genes, and participate in various important physiological processes, including cell proliferation and differentiation 33. Therefore, it is of great significance to identify the ligands and signaling pathways of this orphan receptor. In recent years, many independent studies on RORα have discovered its potential role in human cancers. MiR-18a downregulates RORα through the TNF-α-mediated NF-κB signaling pathway, inhibiting glioma proliferation and tumor development 34. Dp44mT inhibits glioma growth and induces cell apoptosis through the NDRG2-IL6/JAK2/STAT3 signaling pathway mediated by RORα 35. Furthermore, RORα inhibits cell proliferation and has anticancer effects in breast cancer, colon cancer, prostate cancer, endometrial cancer, hepatocellular carcinoma, and other solid tumors 36. RORα mediates the glucose metabolism-reprogramming response to glutamine deprivation and contributes to altered glucose utilization and liver cancer growth 37. However, the role of RORα in ovarian cancer is not yet clear. In our research, we found that PS VII can bind to RORα and enhance its protein stability, thereby inhibiting glycolysis, angiogenesis, and PARP inhibitor resistance in ovarian cancer. Our findings identify RORα as a direct target of PS VII for the first time, clarifying the key role of RORα in glycolysis-induced cancer drug resistance and metastasis. It suggests that PS VII may serve as an RORα activator for targeted therapy in the clinical treatment of ovarian cancer.

Since RORα is an important regulator of gene transcription and can recruit various transcription factors and co-regulators to target gene promoters, we found that RORα can regulate ECM1 transcriptional expression. The extracellular matrix (ECM) plays a crucial role in tumor development, drug resistance, metastasis, and the tumor microenvironment 38. ECM1 is an 85 kDa secreted glycoprotein originally isolated from the mouse MN7 chondrocyte extracellular matrix cell line. The human ECM1 gene is located on chromosome 1q21 and encodes four different splice proteins. Many studies have reported that ECM1 protein has various biological functions and is involved in processes such as embryonic cartilage formation, skin lesions, angiogenesis, and tumor progression 39. Our previous studies have shown that ECM1 regulates the FAK/AKT/GSK3β signaling pathway by interacting with integrin protein β4 (ITGB4) 40. In our research, we found that RORα directly interacts with the ECM1 promoter region and regulates the transcriptional levels of ECM1. ECM1 protein is highly expressed in various malignant epithelial tumors 41. This study is the first to report the binding of RORα to the ECM1 promoter region, promoting ECM1 transcription. Therefore, our research further reveals that ECM1 may play a role as a member of a complex regulatory network in cancer cells and plays an important role in reversing ovarian cancer PARP inhibitor resistance through PS VII.

Furthermore, we have also demonstrated the interaction between ECM1 and VEGFR2. VEGFR2 is primarily expressed in endothelial cells and upregulated in tumor blood vessels 42. During the early stages of tumor growth, the tumor grows slowly and consumes less oxygen and nutrients, resulting in minimal angiogenesis. Without angiogenesis, tumor growth does not exceed 3mm³. As cancer cells grow, the tumor size increases, leading to oxygen and nutrient deprivation within the tumor, which stimulates angiogenic factors in a hypoxic and nutrient-deprived environment 43. Subsequently, tumor cells secrete various inducing factors, leading to neovascularization and providing sufficient oxygen and nutrients to support rapid tumor growth. Several studies have shown that angiogenesis plays a crucial role in invasion, metastasis, and drug resistance in ovarian cancer, and is closely associated with disease progression and prognosis 44. Our research detailedly reveals that PS VII may hinder the binding of ECM1 to VEGFR2 and inhibit the FAK/AKT/GSK3β signaling pathway in ovarian cancer PARP inhibitor-resistant cells, affecting glycolysis, angiogenesis, and other functions, ultimately elucidating the specific mechanism by which it improves resistance.

In conclusion, we found that PS VII reverses PARPi resistance to some extent in ovarian cancer and exhibits better synergistic therapeutic effects when used in combination with PARPi in ovarian cancer treatment. Its potential function may hinder glycolysis and angiogenesis in PARPi resistant ovarian cancer cells, which is associated with PS VII binding and stabilizing RORα expression, further inhibiting ECM1 and blocking the VEGFR2/FAK/AKT/GSK3β signaling pathway. Our research data provides a new targeted therapy approach for clinical ovarian cancer treatment and brings a ray of hope to PARPi-resistant ovarian cancer patients, effectively expanding the application of PARPi in clinical treatment.

## Supplementary Material

Supplementary figures and tables.

## Abbreviations

PS VII: Paris Saponin VII
RORα: retinoid acid receptor-related orphan receptor α
ECM1: extracellular matrix protein 1
VEGFR2: vascular endothelial growth factor receptor 2
CDDP: cisplatin
OS: overall survival
PFS: progression-free survival
ATCC: American Type Culture Collection
IHC: immunohistochemistry
GSEA: Gene Set Enrichment Analysis
qRT-PCR: quantitative reverse transcription polymerase chain reaction
SDS-PAGE: sodium dodecyl sulfate polyacrylamide gel electrophoresis
OCR: oxygen consumption rate
ECAR: extracellular acidification rate
PBS: phosphate-buffered saline
CCK-8: Cell Counting Kit-8
EGFP: enhanced green fluorescent protein
