# Supplementary material for: Paris saponin VII reverses resistance to PARP inhibitors by regulating ovarian cancer tumor angiogenesis and glycolysis through the RORα/ECM1/VEGFR2 signaling axis
Source: Int J Biol Sci. 2024 Apr 15;20(7):2454–75. doi: 10.7150/ijbs.91861 (PMC11077377; doi:10.7150/ijbs.91861)
Supplement: Supplementary file 1 — Supplementary figures and tables. [file ijbsv20p2454s1.pdf]

Supplementary Information

Supplementary Figure 1

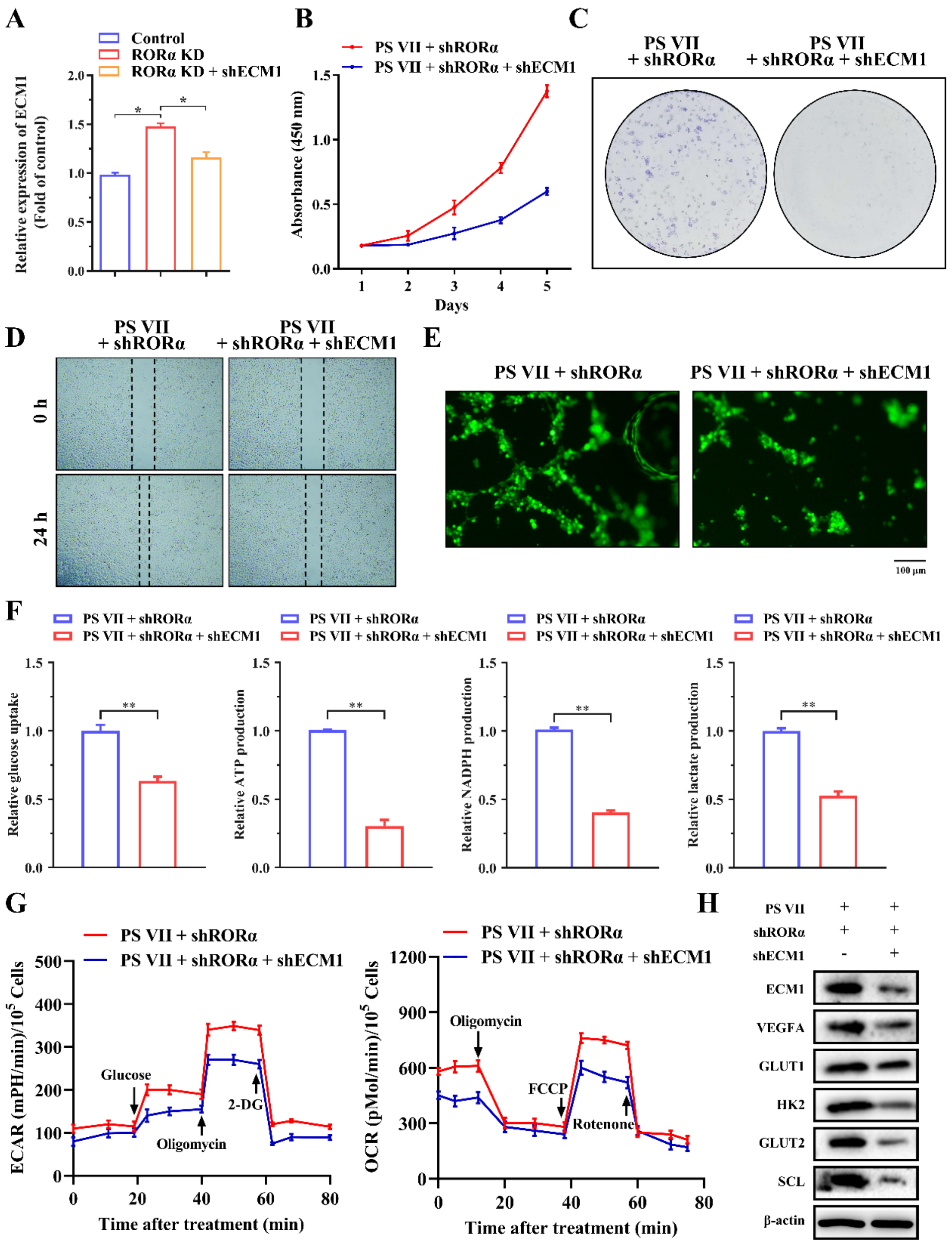

Supplementary Figure 1: Restorative Effect of ECM1 on the Regulation of Glycolysis and Angiogenesis in PARPi-Resistant Cells by PS VII

A. qRT-PCR is used to assess the expression of ECM1 mRNA in shRORα and shECM1

cells.B-C. CCK-8 assay (B) and colony formation assay (C) are performed to evaluate the effect of shRORα and shECM1 on the proliferation rate of PARPi-resistant ovarian cancer cells treated with PS VII.D. Wound healing assay is conducted to examine the effect of shRORα and shECM1 on the migratory and invasive abilities of PARPi-resistant ovarian cancer cells treated with PS VII.E. The impact of shRORα and shECM1 on tube formation in human umbilical vein endothelial cells (HUVECs) is analyzed using conditioned medium derived from PARPi-resistant ovarian cancer cells with shRORα and shECM1 intervention after PS VII treatment.F. The effect of shRORα and shECM1 on glucose uptake, lactate production, ATP production, and NADPH production in PARPi-resistant ovarian cancer cells treated with PS VII is evaluated.G. ECAR and OCR curves are obtained to evaluate the extracellular acidification rate and oxygen consumption rate, respectively, of PARPi-resistant ovarian cancer cells treated with PS VII after shRORα and shECM1 intervention.H. The effect of shRORα and shECM1H. Western blot experiments are performed to investigate the effects of shRORα and shECM1 on PS VII-treated PARPi-resistant ovarian cancer cells in terms of glycolysis, angiogenesis, and metastasis-related proteins. *$p < 0.05$, **$p < 0.01$, ***$p < 0.001$.

Supplementary Figure 2

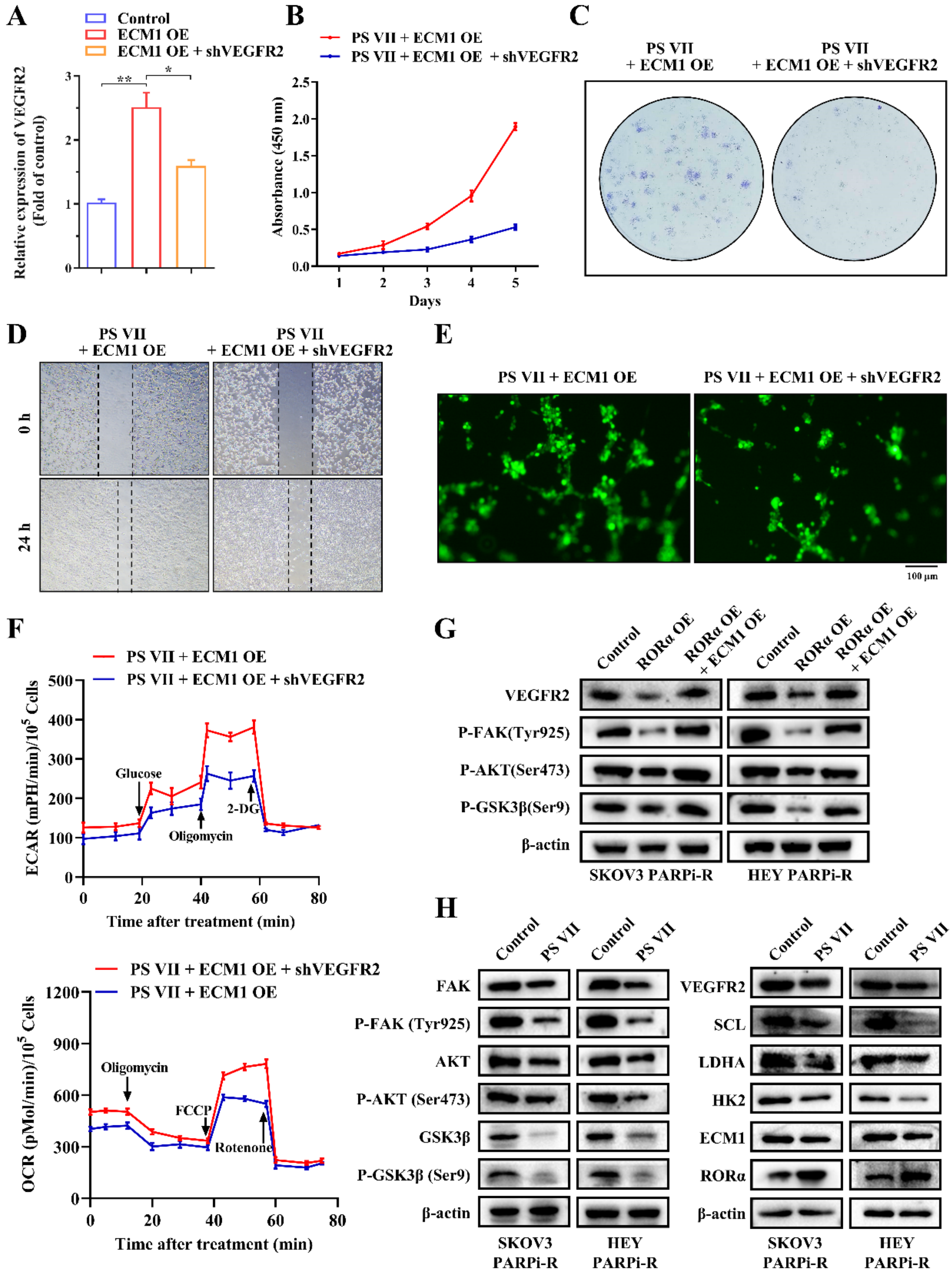

Supplementary Figure 2: Recovery Experiment of PS VII in Reversing PARPi Resistance in Ovarian Cancer Cells through the RORα/ECM1/VEGFR2 Signaling Pathway

A. qRT-PCR is used to detect the expression of VEGFR2 mRNA in shVEGFR2 and

ECM1 OE cells.B-C. CCK-8 assay (B) and colony formation assay (C) are performed to evaluate the effect of shVEGFR2 and ECM1 OE on the proliferation rate of PARPi-resistant ovarian cancer cells treated with PS VII.D. Wound healing assay is conducted to examine the effect of shVEGFR2 and ECM1 OE on the migratory and invasive abilities of PARPi-resistant ovarian cancer cells treated with PS VII.E. The impact of shVEGFR2 and ECM1 OE on tube formation in human umbilical vein endothelial cells (HUVECs) is analyzed using conditioned medium derived from PARPi-resistant ovarian cancer cells with shVEGFR2 and ECM1 OE intervention after PS VII treatment.F. ECAR and OCR curves are obtained to evaluate the extracellular acidification rate and oxygen consumption rate, respectively, of PARPi-resistant ovarian cancer cells treated with PS VII after shVEGFR2 and ECM1 OE intervention.G. Western blot experiments are performed to investigate the effects of shVEGFR2 and ECM1 OE on VEGFR2 and FAK signaling pathway-related protein phosphorylation in PS VII-treated PARPi-resistant ovarian cancer cells.H. The effect of PS VII intervention on FAK signaling pathway-related protein phosphorylation, glycolysis, and angiogenesis-related proteins in PARPi-resistant ovarian cancer cells. $^*p < 0.05$, $^{**}p < 0.01$, $^{***}p < 0.001$.

Supplementary Figure 3

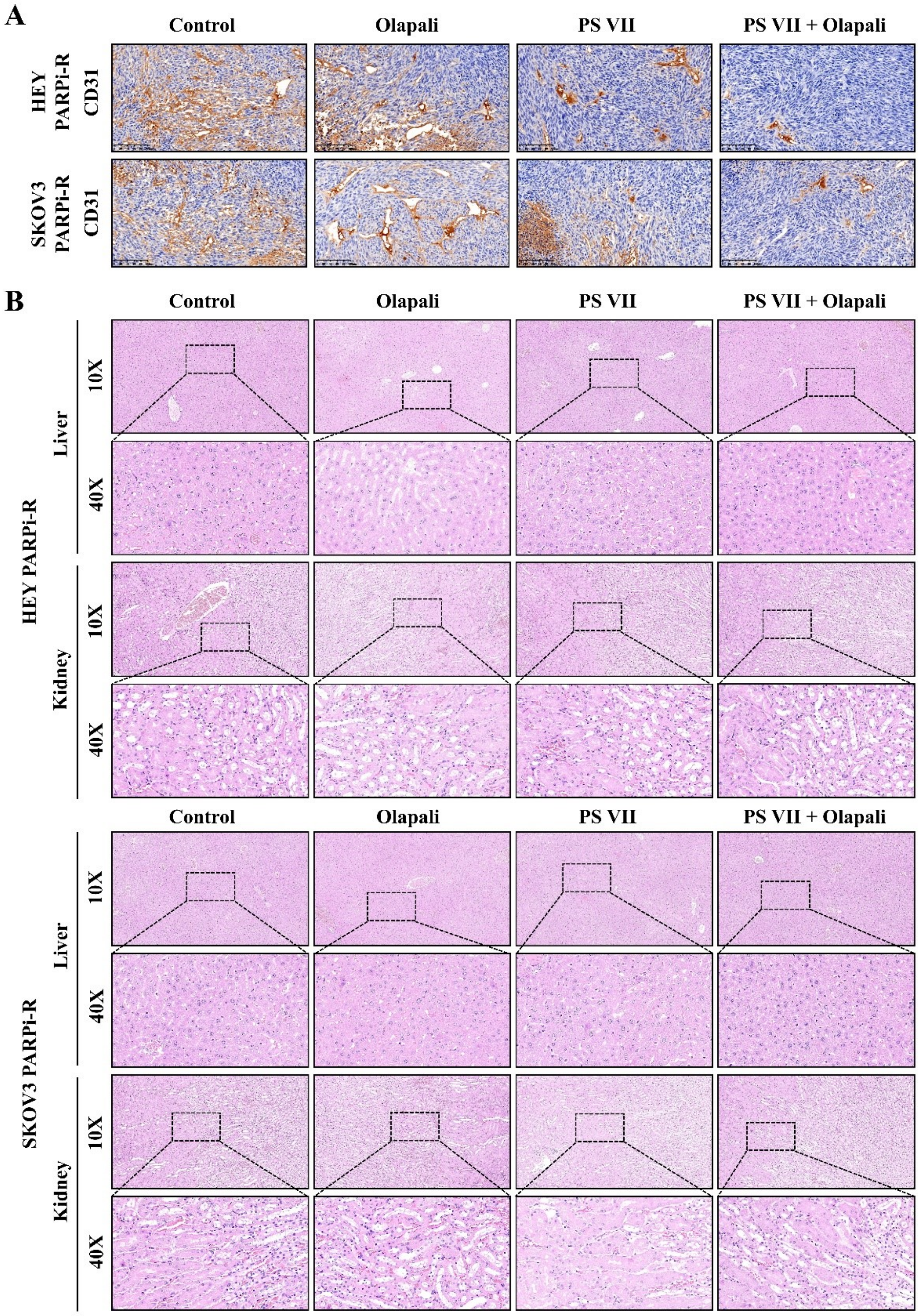

Supplementary Figure 3: Supplementary Figure 3: PS VII Combined with PARPi Can Effectively Inhibit Ovarian Cancer Tumor Angiogenesis with Good Safety

A. Immunohistochemistry (IHC) is performed to evaluate the protein levels of CD31 in the tumor tissues of each group (all at 100× and 400× magnification).B. Comparison of

H&E staining of liver and kidney tissues is performed among different groups.

Supplementary Figure 4

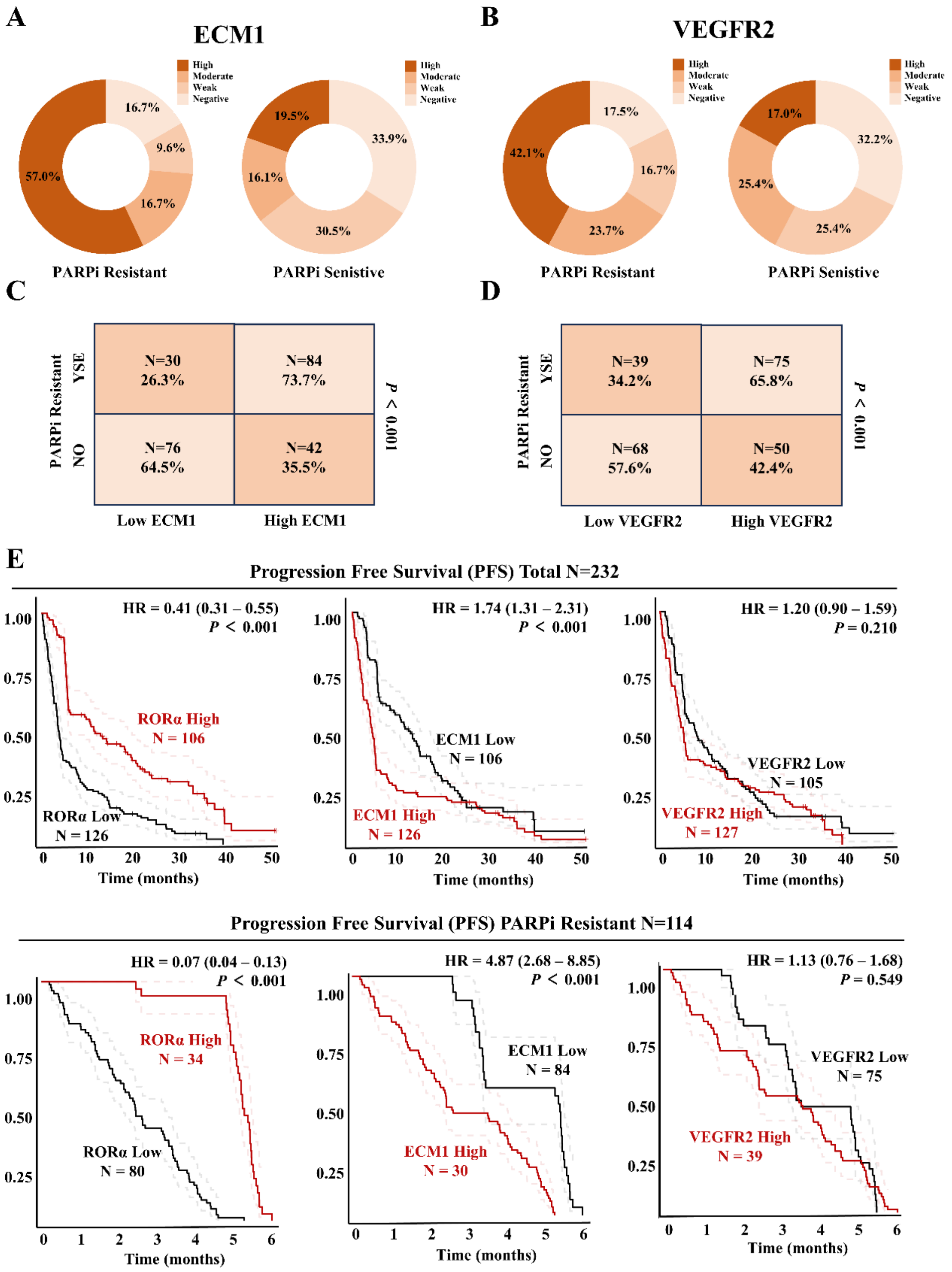

Supplementary Figure 4: Association of ECM1, VEGFR2 with PARPi Resistance and Prognosis in Ovarian Cancer

A-D. Based on the IHC staining scores, ovarian cancer patients are divided into high and low expression groups for ECM1 and VEGFR2. H represents high; L represents

low. The correlation between ECM1 and VEGFR2 expression levels and PARPi resistance is determined.E. Kaplan-Meier analysis is performed to investigate the relationship between RORα, ECM1, VEGFR2, and progression-free survival in all ovarian cancer patients and PARPi-resistant ovarian cancer patients.

Supplementary Figure 5

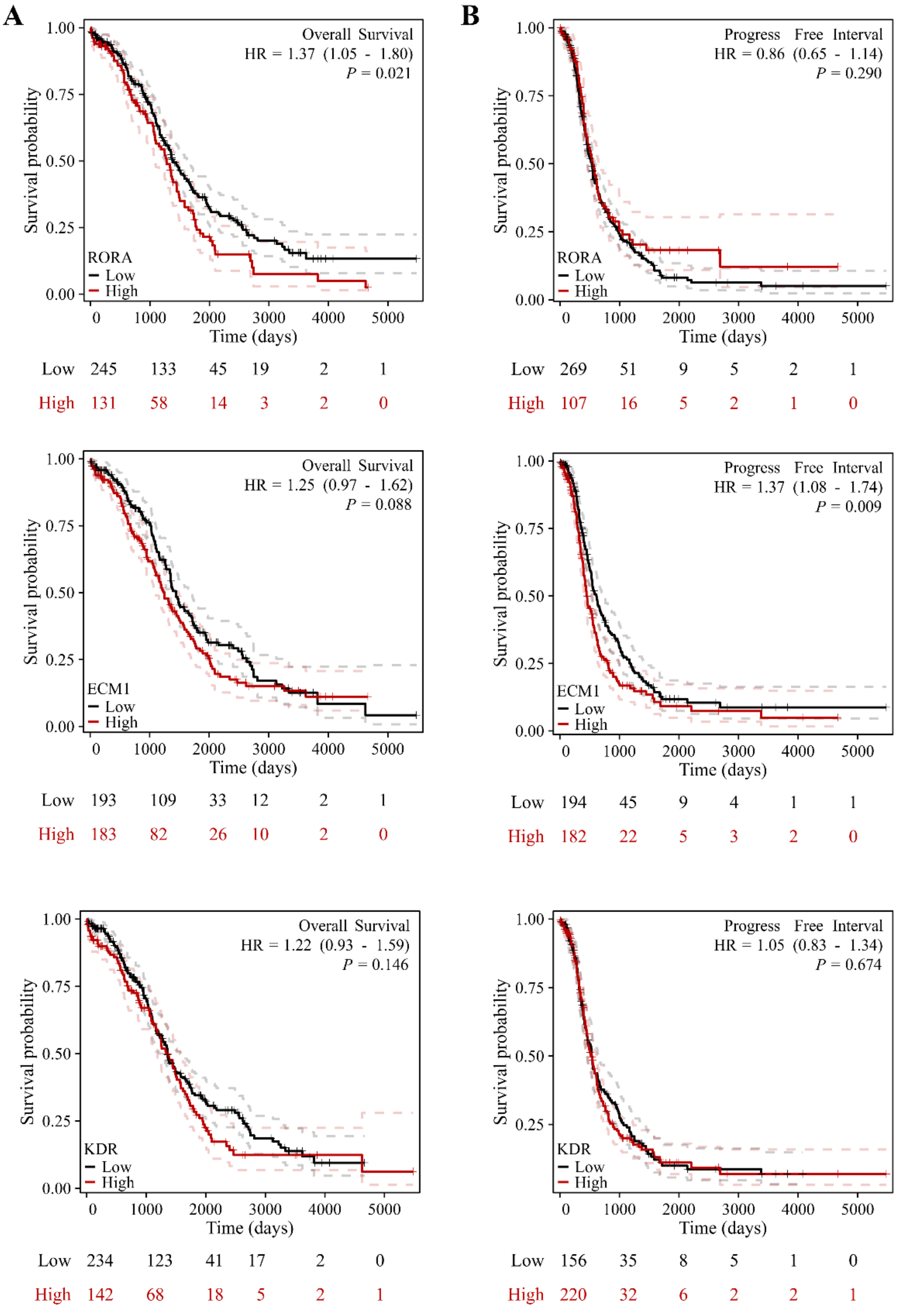

Supplementary Figure 5: Correlation of RORα/ECM1/VEGFR2 with Prognosis in Ovarian Cancer in TCGA Database

A. Kaplan-Meier analysis is performed to investigate the relationship between RORα/ECM1/VEGFR2 and overall survival in ovarian cancer patients in the TCGA

database.B. Kaplan-Meier analysis is performed to investigate the relationship between RORα/ECM1/VEGFR2 and progression-free survival in ovarian cancer patients in the TCGA database.

Supplementary Table 1. The association between clinicopathological characteristics and overall survival in ovarian cancer patients

Prognostic factors	Patients N (%)	Univariate		Multivariate
		P [a]	HR (95 % CI) [b]	*P* [b]
**All patients**	232 (100)			
**Age (years)**				
< 60 (median)	111 (47.9)			
≥ 60 (median)	121 (52.1)	0.255		
**Ascites**				
Absence	70 (30.1)			
Present	162 (69.9)	0.165		
**BRCA mutation**				
BRCA1	86 (37.0)			
BRCA2	146 (63.0)	0.074		
**RORα expression status**				
Positive	106 (45.7)		1.000	
Negative	126 (54.3)	**< 0.001**	0.586 (0.437-0.632)	**< 0.001**
**ECM1 expression status**				
Negative	106 (45.7)		1.000	
Positive	126 (54.3)	**0.005**	1.315 (1.156-1.413)	**0.028**
**VEGFR2 expression status**				
Negative	107 (46.1)		1.000	
Positive	125 (53.9)	**0.009**	1.415 (1.256-1.592)	**0.041**
**Chemotherapeutic response**				
PARPi sensitive	118 (50.9)		1.000	
PARPi resistant	114 (49.1)	**< 0.001**	3.840 (2.787-5.289)	**< 0.001**

Kanplan-Meier survival analysis and Cox proportional hazards regression analysis

[a]without adjustment

[b]with adjustment for RORα, ECM1, VEGFR2 expressioin status and chemotherapeutic response

Supplementary Table 2. **The relationship between clinicopathological characteristics and RORα, ECM1, VEGFR2 expression in ovarian cancer patients**

Prognostic factors	RORα-low (%)	RORα-high (%)	P [a]	ECM1-low (%)	ECM1-high (%)	P [a]	VEGFR2-low (%)	VEGFR2-high (%)	P [a]
**Age**			**0.003**			**0.025**			**0.004**
≤60 (median)	96 (54.2)	111 (56.1)		108 (52.4)	99 (58.6)		178 (57.6)	29 (43.9)	
> 60 (median)	81 (45.8)	87 (43.9)		98 (47.6)	70 (41.4)		131 (42.4)	37 (56.1)	
**Ascites**			**< 0.001**			**< 0.001**			**< 0.001**
Absence	16 (9.0)	35 (17.7)		36 (17.5)	15 (8.9)		42 (13.6)	9 (13.6)	
Present	161 (91.0)	163 (82.3)		170 (82.5)	154 (91.1)		267 (86.4)	57 (86.4)	
**BRCA mutation**			**< 0.001**			**< 0.001**			**< 0.001**
BRCA1	141 (79.7)	185 (93.4)		183 (88.8)	143 (84.6)		267 (86.4)	59 (89.4)	
BRCA2	36 (20.3)	13 (6.6)		23 (11.2)	26 (15.4)		42 (13.6)	7 (10.6)	
**Chemotherapeutic response**			**< 0.001**			**< 0.001**			**< 0.001**
PARPi sensitive	66 (37.3)	96 (48.5)		98 (47.6)	64 (37.9)		138 (44.7)	24 (36.4)	
PARPi resistant	111 (62.7)	102 (51.5)		108 (52.4)	105 (62.1)		171 (55.3)	42 (63.6)	
**Recurrence**			**< 0.001**			**0.005**			0.635
Absence	48 (27.1)	86 (43.4)		83 (40.3)	51 (30.2)		115 (37.2)	19 (28.8)	
Present	129 (72.9)	112 (56.6)		123 (59.7)	118 (69.8)		194 (62.8)	47 (71.2)	
**Survival situation**			**< 0.001**			**0.011**			0.270
survival	48 (27.1)	86 (43.4)		83 (40.3)	51 (30.2)		115 (37.2)	19 (28.8)	
death	129 (72.9)	112 (56.6)		123 (59.7)	118 (69.8)		194 (62.8)	47 (71.2)	

Two-sided χ^2 test or Fisher's exact test for distributions between negative and positive expression of RORα, ECM1, VEGFR2

Supplementary Table 3. List of Primers Sequences

Primer Name	Primer Sequences (5′ - 3′)
**qRT: 18S RNA**	F: AGGAATTCCCAGTAAGTGCG<br>R: GCCTCACTAAACCATCCAA
**qRT: RORα**	F: CTTGCCGTAGGGATGTCTCG<br>R: GAAGTTCCGTCAGCCCGTT
**qRT: ECM1**	F: AGCACCCCAATGAACAGAAGG<br>R: CTGCATTCCAGGACTCAGGTT
**qRT: VEGFR2**	F:TGGTGGTGTGGATGGAGAGT<br>R:TCTGGAGACTGGAGGGAGTC
**RORα-OE**	F: TGCTCTAGAATGAATGAGGGGGCCCCAGGAGACAGTGAC<br>R: CCGGAATTCTTACCCATCAATTTGCATTGCTGGCTC
**ECM1-OE**	F: TGCTCTAGAATGGGGACCACAGCCAGAGCAGCCTTGGTC<br>R: CCGGAATTCTCATTCTTCCTTGGGCTCAGAGGTGGAGC
**ECM1-WT promoter**	F: TGCTCTAGAGCCCATTCACTAACCCAAAG<br>R: CCGGAATTCAAAGTCAGCAGCAGACCCATA
**ECM1-MUT promoter**	F: TCAGTTTCTCTCGCATGCCTTAAGA<br>R: AACATTCTTAAGGCATGCGAGAGA
